# Pathological Tumor–Nodal Response Patterns After Neoadjuvant Treatment for Rectal Cancer: Prognostic Impact of Residual ypN-Positive Disease

**DOI:** 10.3390/cancers18203352

**Published:** 2026-10-16

**Authors:** Yusuf Emre Aytin, Sezer Berkay Gorca

**Affiliations:** Department of General Surgery, Faculty of Medicine, Trakya University, Edirne 22030, Türkiye; sberkaygorca@trakya.edu.tr

**Keywords:** rectal cancer, neoadjuvant therapy, pathological complete response, residual nodal disease, lymphatic metastasis, recurrence, prognosis, ypT0N+

## Abstract

**Background/Objectives:** Pathological response to neoadjuvant treatment in rectal cancer may differ between the primary tumor and regional lymph nodes, and the prognostic implications of this discordance remain incompletely defined. We evaluated oncological outcomes according to combined pathological tumor–nodal response patterns, with particular emphasis on complete primary-tumor regression with residual ypN-positive disease. **Methods:** A total of 215 patients with nonmetastatic rectal adenocarcinoma who received neoadjuvant treatment followed by curative-intent resection were retrospectively analyzed. Patients were classified as ypT0N0 (*n* = 34), ypT0N+ (*n* = 13), residual primary tumor/ypN0 (*n* = 119), or residual primary tumor/ypN+ (*n* = 49). The primary endpoint was time to recurrence (TTR). Kaplan–Meier, Cox, Firth penalized Cox, competing-risk, and sensitivity analyses were performed. **Results:** Median follow-up was 70.0 months. Recurrence occurred in 2/34 (5.9%), 6/13 (46.2%), 17/119 (14.3%), and 18/49 (36.7%) patients, respectively. Estimated 5-year recurrence-free probabilities were 90.1%, 53.8%, 81.6%, and 46.3%, respectively (log-rank *p* < 0.0001). Compared with ypT0N0, recurrence hazard was significantly higher for ypT0N+ (adjusted hazard ratio 7.56, 95% confidence interval 1.49–38.40; *p* = 0.015) and residual primary tumor/ypN+ (adjusted hazard ratio 7.10, 95% confidence interval 1.64–30.83; *p* = 0.009). In an analysis restricted to patients with baseline cT3N1 disease, the association for ypT0N+ remained evident (adjusted hazard ratio 6.34, 95% confidence interval 1.19–33.79; *p* = 0.031). Competing-risk analyses were concordant, and all documented recurrences in the ypT0N+ group involved distant metastasis. **Conclusions:** In this cohort, residual ypN-positive disease despite complete pathological regression of the primary tumor was associated with an increased risk of recurrence. Given the small number of patients with ypT0N+ disease, these findings should be interpreted cautiously and require validation in larger independent cohorts.

## Simple Summary

Neoadjuvant treatment can completely eliminate the primary rectal tumor while residual cancer remains in regional lymph nodes or tumor deposits. This study examined whether considering primary-tumor and nodal response separately improves prognostic assessment after neoadjuvant treatment. A total of 215 patients with nonmetastatic rectal cancer who underwent curative-intent surgery were retrospectively evaluated and classified into four pathological tumor–nodal response groups. Patients with complete regression of the primary tumor and no residual nodal disease had the most favorable outcomes. In contrast, patients with complete primary-tumor regression but persistent nodal disease experienced substantially higher risks of recurrence and distant metastasis. Because only 13 patients had this uncommon ypT0N+ response pattern, these findings should be interpreted cautiously and confirmed in larger studies. These findings suggest that complete regression of the primary tumor should not be interpreted as a uniformly favorable response when nodal disease remains and support separate consideration of nodal status in postoperative risk stratification.

## 1. Introduction

Neoadjuvant treatment has become an integral component of multidisciplinary management for locally advanced rectal cancer, with treatment response emerging as an important determinant of subsequent oncological outcomes [1]. The magnitude of pathological regression after preoperative therapy varies considerably, ranging from extensive residual disease to complete eradication of viable tumor [2,3]. At the favorable end of this spectrum, pathological complete response has consistently been associated with lower recurrence rates and prolonged survival, reinforcing its value as both a prognostic marker and a clinically meaningful treatment endpoint [4,5,6]. Nevertheless, response to neoadjuvant therapy is not necessarily uniform across the primary tumor and regional lymphatic compartment. Consequently, assessment of treatment efficacy based predominantly on regression within the rectal wall may incompletely characterize the residual oncological burden after neoadjuvant therapy.

Complete regression of the primary rectal tumor does not necessarily indicate complete eradication of locoregional disease. A small but clinically relevant proportion of patients classified as ypT0 after neoadjuvant treatment continue to harbor residual ypN-positive disease, defining a distinct ypT0N+ phenotype rather than a true pathological complete response [7,8]. Multicenter, population-based, and recent ypT0-specific cohort data indicate that persistent nodal involvement in this setting is associated with substantially less favorable disease control and survival than ypT0N0, suggesting that complete regression of the primary tumor cannot, in isolation, be regarded as a uniformly favorable prognostic state [7,8,9,10,11]. More broadly, studies evaluating discordant tumor and nodal responses after neoadjuvant therapy have shown that residual nodal disease may outweigh an otherwise favorable primary-tumor response in determining oncological outcome [12]. This discordance also creates a clinically relevant staging challenge, as the prognostic position of ypT0N+ disease relative to other post-neoadjuvant pathological states remains incompletely defined [8,9].

The prognostic relevance of nodal response extends beyond the uncommon ypT0N+ phenotype. Across contemporary rectal cancer cohorts, postoperative ypN status has emerged as a major determinant of long-term outcome, with patients who achieve nodal downstaging to ypN0 demonstrating substantially more favorable survival than those with persistent nodal disease [13,14]. This relationship appears to remain clinically meaningful irrespective of the degree of primary-tumor regression, supporting the concept that lymphatic response represents a distinct component of treatment sensitivity rather than a simple surrogate of tumor-bed response [13,15,16]. Moreover, accurate characterization of residual nodal disease after neoadjuvant therapy is important not only for prognostication but also for postoperative risk stratification, because nodal positivity identifies a population with greater potential for subsequent systemic failure [15,17]. Nevertheless, most post-neoadjuvant prognostic frameworks continue to emphasize conventional ypTNM categories or pathological complete response as composite endpoints, leaving the interaction between complete versus incomplete primary-tumor regression and residual nodal status comparatively underexplored [17]. A response-based classification that preserves information from both pathological compartments may therefore provide additional prognostic information beyond assessment of the primary tumor alone [16,18].

Within this context, patients with complete primary-tumor regression but persistent nodal disease represent a clinically distinct subgroup whose long-term risk may not be adequately captured by conventional response categories. Rather than examining ypT0N+ disease in isolation, we evaluated four mutually exclusive pathological tumor–nodal response patterns to determine whether residual nodal disease modifies the prognostic meaning of complete or incomplete primary-tumor regression. Accordingly, oncological outcomes were compared across patients with complete primary-tumor regression with or without residual nodal disease and those with residual primary tumor according to nodal status.

## 2. Materials and Methods

### 2.1. Study Design and Patient Population

This retrospective cohort study was conducted at the Department of General Surgery, Trakya University Faculty of Medicine, Edirne, Türkiye. Consecutive patients with histologically confirmed rectal adenocarcinoma who underwent surgery between 1 January 2018 and 15 August 2023 were screened for eligibility. The upper limit of the inclusion period was selected to ensure at least 36 months of potential follow-up for the most recently operated patients before final outcome ascertainment.

Patients were eligible if they had nonmetastatic rectal adenocarcinoma at initial diagnosis, received neoadjuvant treatment, subsequently underwent curative-intent rectal resection, and had sufficient postoperative follow-up to permit reliable ascertainment of oncological outcomes. Patients were excluded if they had not received neoadjuvant treatment, had distant metastatic disease at initial presentation (cM1), did not undergo curative-intent rectal resection, or lacked sufficient postoperative oncological follow-up to permit reliable ascertainment of recurrence and survival outcomes.

**Figure 1:**
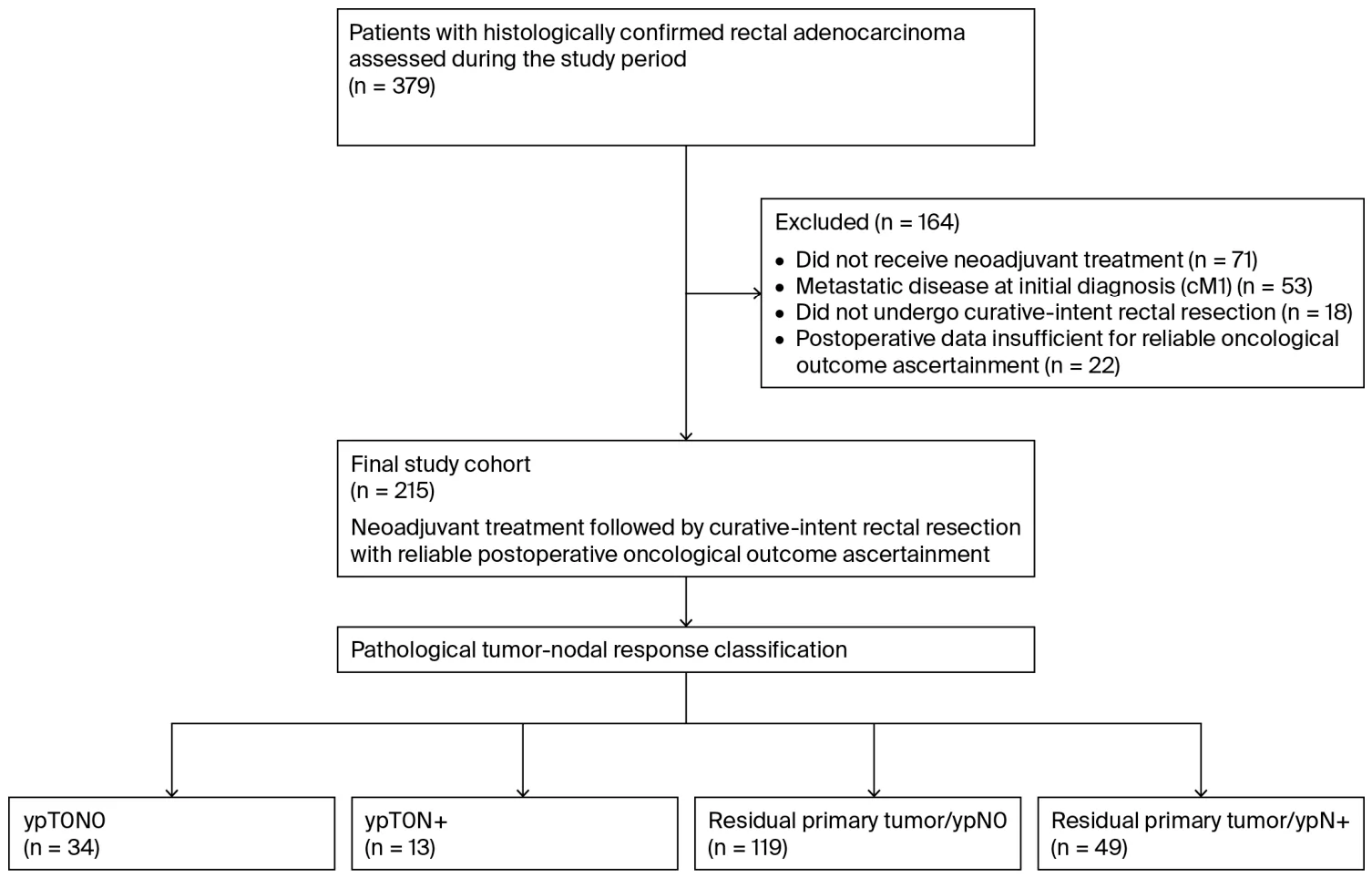
Patient flow diagram and pathological tumor–nodal response classification. Patients with histologically confirmed rectal adenocarcinoma were assessed for eligibility, and exclusions were applied according to neoadjuvant treatment status, metastatic disease at presentation, curative-intent rectal resection, and availability of reliable postoperative oncological outcome data. The final cohort was classified into four pathological tumor–nodal response groups: ypT0N0, ypT0N+, residual primary tumor/ypN0, and residual primary tumor/ypN+.

A total of 379 patients were assessed during the study period. Of these, 71 had not received neoadjuvant treatment, 53 had metastatic disease at initial diagnosis, and 18 did not undergo curative-intent resection. Twenty-two patients were excluded because reliable postoperative oncological outcome ascertainment was not possible. These patients either did not return for postoperative follow-up or attended only an initial post-discharge surgical visit before continuing oncological care at another institution, with no subsequent clinical follow-up data available in the accessible medical records. The final study cohort therefore comprised 215 patients (Figure 1).

Clinical, treatment-related, operative, pathological, and follow-up data were retrospectively retrieved from institutional medical records, operative reports, pathology reports, oncology records, and follow-up documentation. Pretreatment clinical T and N categories were retrospectively abstracted from the baseline pelvic MRI reports available in the institutional medical records. No retrospective radiological re-review of the original MRI images was performed. Data collection was performed using a predefined study dataset before statistical analysis.

### 2.2. Neoadjuvant Treatment and Surgical Management

Neoadjuvant treatment was administered according to multidisciplinary evaluation and contemporary institutional practice. Long-course chemoradiotherapy typically consisted of a total radiation dose of 45.0–50.4 Gy delivered in 25–28 fractions with concurrent capecitabine-based radiosensitizing chemotherapy. Short-course radiotherapy consisted of 25.0 Gy delivered in five fractions and was administered without concurrent chemotherapy. Among the 215 patients, 168 (78.1%) received long-course chemoradiotherapy and 47 (21.9%) received short-course radiotherapy. The specific patient-level rationale for selecting one neoadjuvant approach over the other was not systematically captured in the retrospective records and therefore could not be reliably reconstructed. No patient in the analytic cohort received total neoadjuvant therapy or a separate induction or consolidation systemic chemotherapy regimen. Treatment type was recorded as a categorical variable and incorporated into the analyses because the neoadjuvant strategy could potentially influence pathological response and subsequent oncological outcomes. Neoadjuvant treatment was considered completed when the planned preoperative treatment course was completed as documented in the oncology records; patients in whom the planned course was prematurely discontinued were classified as having incomplete neoadjuvant treatment.

Following completion of neoadjuvant treatment, patients underwent curative-intent rectal resection using total mesorectal excision principles. The operative procedure was categorized as low anterior resection, ultra-low anterior resection, or abdominoperineal resection. Surgical approach, presence of colorectal anastomosis, and postoperative stoma status were also recorded.

The interval from neoadjuvant treatment to surgery was calculated in days from the actual final date of the administered neoadjuvant treatment course, as documented in the oncology records, to the date of surgery and was analyzed as a continuous variable.

Postoperative systemic therapy was determined according to pathological findings, patient condition, multidisciplinary assessment, and prevailing oncological practice. Adjuvant chemotherapy regimens included FOLFOX (folinic acid, fluorouracil, and oxaliplatin), CAPOX/XELOX (capecitabine and oxaliplatin), or capecitabine monotherapy. Detailed information regarding completion of the planned postoperative chemotherapy course was not consistently available in the retrospective records and was therefore not analyzed. Because the indication for postoperative chemotherapy was inherently influenced by postoperative pathological findings, adjuvant chemotherapy was considered a post-exposure treatment variable and was not included in the primary multivariable TTR model in order to minimize treatment-by-indication bias and overadjustment.

### 2.3. Pathological Assessment

Resection specimens were evaluated by the institutional pathology service according to routine standardized pathological assessment. Pathological staging was performed according to the eighth edition of the American Joint Committee on Cancer (AJCC) TNM classification [19]. Pathological variables included ypT category, ypN category, number of examined and metastatic lymph nodes, circumferential resection margin status, histological subtype, tumor differentiation, lymphovascular invasion, perineural invasion, venous invasion, tumor budding, and tumor deposits when reported.

The number of examined lymph nodes was recorded as a continuous variable and additionally categorized as <12 or ≥12 nodes. No minimum lymph-node yield was required for classification as ypN0; the ≥12-node threshold was used only for descriptive categorization and sensitivity analysis. Regional nodal status was categorized as ypN0, ypN1, or ypN2. Cases classified as ypN1c on the basis of tumor deposits in the absence of metastatic regional lymph nodes were considered within the ypN-positive pathological category for the principal tumor–nodal response classification. Following neoadjuvant treatment, acellular mucin pools without viable tumor cells were not considered residual primary tumor or nodal disease for ypTN staging, and tumor deposits were classified as ypN1c only when viable tumor cells were present.

Circumferential resection margin (CRM) status was abstracted directly from the radial surgical margin assessment documented in the final pathology report and was categorized as negative or positive. Cases explicitly reported as having a positive/involved radial surgical margin were classified as CRM-positive, whereas those reported as having a negative radial surgical margin were classified as CRM-negative. Because the exact tumor-to-margin distance in millimeters was not consistently available, no retrospective distance-based reclassification was performed.

Histological subtype was categorized as conventional adenocarcinoma, mucinous adenocarcinoma, or signet-ring cell carcinoma. Tumor differentiation was categorized as well, moderately, or poorly differentiated. For patients with ypT0 disease, in whom no residual primary tumor was present in the resection specimen, histological subtype and differentiation were derived from the pretreatment diagnostic biopsy. For patients with residual primary tumor, these variables were obtained from the resection specimen.

Lymphovascular invasion, perineural invasion, venous invasion, and tumor budding were evaluated only when residual primary tumor was present. These variables were therefore considered not assessable for comparative purposes in patients with ypT0 disease and were not imputed.

### 2.4. Assessment of Primary Tumor Regression

Histopathological primary tumor regression was assessed using the modified Ryan tumor regression grading system [20], as documented in the pathology reports: grade 0, complete response with no viable cancer cells; grade 1, near-complete response with single cells or rare small groups of cancer cells; grade 2, partial response with residual cancer showing evident tumor regression but more than single cells or rare small groups; and grade 3, poor or no response with extensive residual cancer and no evident tumor regression.

Importantly, the modified Ryan grade reflects treatment response within the primary tumor bed and does not incorporate residual regional nodal disease. Consequently, tumor regression grade was not used to define the principal exposure groups in the present study and was not included as a covariate in the primary multivariable model, because doing so would introduce substantial structural overlap with postoperative pathological tumor status.

### 2.5. Definition of Pathological Tumor–Nodal Response Groups

The principal exposure of interest was the combined pathological status of the primary rectal tumor and regional lymph nodes after neoadjuvant treatment.

Patients were classified into four mutually exclusive pathological tumor–nodal response groups:

ypT0N0: complete pathological regression of the primary tumor with no residual regional nodal disease;

ypT0N+: complete pathological regression of the primary tumor with residual ypN-positive disease;

Residual primary tumor/ypN0: residual primary tumor (ypTis–ypT4) with no regional nodal disease;

Residual primary tumor/ypN+: residual primary tumor (ypTis–ypT4) with residual ypN-positive disease.

This classification was selected to distinguish complete eradication of the primary tumor from pathological complete response and, specifically, to determine whether residual nodal disease modifies the prognostic significance of complete primary tumor regression.

Pathological complete response was therefore represented exclusively by the ypT0N0 group. Patients with ypT0N+ disease were not considered to have achieved pathological complete response despite complete regression of the primary tumor.

### 2.6. Follow-Up and Definition of Oncological Outcomes

Postoperative surveillance was performed according to institutional oncological practice. Follow-up information was obtained from outpatient records, oncology documentation, hospital databases, imaging reports, and available clinical records. Vital status information was updated through September 2026.

The primary oncological endpoint was time to recurrence (TTR). TTR was calculated from the date of surgery to the first documented local or distant recurrence. Patients without documented recurrence were censored at the date of their last available oncological follow-up. For Kaplan–Meier and Cox analyses, death in the absence of documented recurrence was treated as a censoring event; in competing-risk analyses, death without recurrence was treated as a competing event.

Time to distant metastasis was calculated from the date of surgery to the first documented distant metastatic event. For Kaplan–Meier analyses, patients who died without documented distant metastasis were treated as censored at the date of death, whereas patients remaining alive without distant metastasis were censored at their last available oncological follow-up. In competing-risk analyses, death without distant metastasis was treated as a competing event.

Time to local recurrence was calculated from the date of surgery to the first documented pelvic/local recurrence, with censoring at the last oncological follow-up among patients without local recurrence.

Overall survival (OS) was calculated from the date of surgery to death from any cause. Patients alive at the end of follow-up were censored at the most recent date on which vital status was confirmed.

For patients who subsequently experienced both local and distant recurrence, the first documented recurrence determined the TTR event date, whereas the first documented distant metastatic event determined the time-to-distant-metastasis event date.

Early postoperative mortality was additionally characterized at 30 and 90 days. Because early postoperative deaths could influence estimates of long-term OS independently of tumor biology, an exploratory sensitivity analysis was performed after excluding patients who died within 90 days after surgery.

### 2.7. Statistical Analysis

Distributional properties of continuous variables were evaluated by visual inspection together with the Shapiro–Wilk test. Variables showing an approximately normal distribution are summarized as mean ± standard deviation, while those with non-normal distributions are presented as median with interquartile range. Categorical data are reported as frequencies and percentages.

For comparisons across the four pathological tumor–nodal response categories, normally distributed continuous variables were analyzed by one-way analysis of variance, whereas the Kruskal–Wallis test was used for variables that did not meet normality assumptions. Variance homogeneity was examined where relevant. Associations involving categorical variables were assessed with Pearson’s chi-square test, with Fisher’s exact test used when expected cell counts were insufficient for chi-square testing.

Lymphovascular invasion, perineural invasion, venous invasion, and tumor budding were considered evaluable only in specimens containing residual primary tumor. Accordingly, comparisons of these variables were restricted to the residual primary tumor/ypN0 and residual primary tumor/ypN+ groups. Patients with ypT0 disease were not classified as negative for these pathological features.

Follow-up duration was summarized using the reverse Kaplan–Meier approach. Kaplan–Meier methods were used to estimate time-to-event outcomes, and overall differences among the four pathological tumor–nodal response groups were tested with the log-rank test. Estimates at 3 and 5 years were reported with 95% confidence intervals when applicable. For pairwise group comparisons, multiplicity was controlled using the Holm adjustment.

Recurrence and distant metastasis were also examined within a competing-risk framework. Cumulative incidence functions were estimated with death before the corresponding oncological event treated as the competing event. Global differences between response groups were evaluated with Gray’s test, and Holm-adjusted *p* values were used for pairwise comparisons.

Associations with TTR were examined using Cox proportional hazards regression. Univariable models were first fitted for clinically relevant candidate variables. The primary adjusted model included pathological tumor–nodal response group, neoadjuvant treatment type, age, and baseline serum carcinoembryonic antigen (CEA). This limited covariate set was selected to retain the prognostic contribution of tumor–nodal response while reducing model instability and avoiding adjustment for variables occurring downstream of the pathological response itself. Because the variables included in this model had no missing values, the adjusted analysis included the entire cohort of 215 patients.

Given the small number of recurrence events in the ypT0N0 reference group, robustness of the primary estimates was further examined with Firth penalized Cox regression using the same covariate set. Adjusted comparisons between clinically relevant response groups were additionally derived by changing the model reference category while leaving the remaining covariates unchanged. Further sensitivity analyses assessed whether the principal estimates were maintained after adjustment for pretreatment clinical T category and when baseline CEA was entered on the natural-logarithmic scale. Pretreatment clinical N category was not included in these adjusted sensitivity models because overlap across the pathological response groups was limited. To further address potential confounding by pretreatment clinical stage, the primary Cox model was also repeated after restricting the cohort to patients with pretreatment cT3N1 disease, representing the largest baseline-stage stratum shared across all four pathological response groups. This restricted analysis retained the same covariate structure as the primary model.

Postoperative pathological variables that were closely related to the exposure definition, including CRM status and tumor differentiation, were therefore omitted from the primary adjusted model. To assess whether CRM positivity could account for the observed associations, an additional sensitivity analysis repeated the primary Cox model after excluding all CRM-positive patients. Adjuvant chemotherapy was also not entered into this model because it was initiated after pathological assessment and was strongly determined by adverse postoperative pathological findings, creating a substantial risk of confounding by indication.

Cox model results are expressed as hazard ratios (HRs) or adjusted hazard ratios (aHRs), each with 95% confidence intervals. The proportional hazards assumption was examined using Schoenfeld residuals both for individual covariates and at the global model level.

For time to distant metastasis, Kaplan–Meier estimates and log-rank testing were complemented by competing-risk cumulative incidence analysis with Gray’s test, in which death without distant metastasis was treated as the competing event. Because this was a secondary endpoint and the number of distant metastatic events was relatively limited, a separate multivariable Cox model was not fitted.

Overall survival was analyzed with Kaplan–Meier estimation and log-rank testing. Adjusted associations with OS were assessed in a Cox model containing pathological tumor–nodal response group, neoadjuvant treatment type, age, and baseline serum CEA. Schoenfeld residuals were again used to examine the proportional hazards assumption. To explore whether early postoperative deaths influenced longer-term survival estimates, the same multivariable model was repeated after excluding patients who died within 90 days of surgery.

Because only seven local recurrences were observed, local recurrence was not modeled with multivariable Cox regression, thereby avoiding unstable estimates from an inadequately supported model. Local recurrence rates according to neoadjuvant treatment type were compared using Fisher’s exact test because of the small number of events.

Within the ypT0N+ subgroup, recurrence and distant metastasis were also summarized according to the form of residual ypN-positive disease: metastatic regional lymph-node involvement (ypN1a/ypN1b) or ypN1c tumor deposits. Owing to the small numbers in these exploratory subgroups, no formal inferential comparison or separate prognostic model was undertaken.

A further sensitivity analysis addressed potential nodal understaging associated with limited lymph-node retrieval by repeating the primary multivariable TTR model in the subgroup with a lymph-node yield of ≥12.

All statistical tests were two-sided, with *p* < 0.05 defining statistical significance. Analyses were conducted in jamovi version 2.3.21.0. Proportional hazards diagnostics were performed in R version 4.1.2 through the jamovi Rj interface and were additionally checked in R version 4.6.1 using Google Colaboratory. Firth penalized Cox regression and competing-risk analyses were implemented in R with the coxphf and cmprsk packages, respectively, with confirmatory Firth analyses performed using coxphf version 1.13.4. Median follow-up by the reverse Kaplan–Meier method was calculated in Python version 3.13.15 using lifelines version 0.30.3. Kaplan–Meier estimates, numbers at risk, and the final survival figures were also generated or independently cross-checked in Python within Google Colaboratory.

### 2.8. Ethical Approval

The study was conducted in accordance with the principles of the Declaration of Helsinki and applicable institutional standards for retrospective clinical research. The study protocol was approved by the Trakya University Faculty of Medicine Dean’s Office Non-Interventional Scientific Research Ethics Committee, Edirne, Türkiye (TUTF-GOBAEK 2026/429; Decision No. 16/14; approval date: 14 September 2026). Individual informed consent was not obtained because the study was retrospective and used routinely collected clinical data; the ethics committee reviewed and approved the study without an informed consent form. Patient data were analyzed in de-identified form, and confidentiality was maintained throughout the study.

## 3. Results

### 3.1. Study Cohort and Pathological Tumor–Nodal Response Groups

Among 379 patients with histologically confirmed rectal adenocarcinoma assessed during the study period, 164 were excluded because they had not received neoadjuvant treatment (*n* = 71), had metastatic disease at initial diagnosis (*n* = 53), did not undergo curative-intent rectal resection (*n* = 18), or lacked postoperative data permitting reliable oncological outcome ascertainment (*n* = 22). The final study cohort therefore comprised 215 patients (Figure 1).

According to the pathological status of the primary tumor and regional lymph nodes after neoadjuvant treatment, patients were classified into four groups: ypT0N0 (*n* = 34, 15.8%), ypT0N+ (*n* = 13, 6.0%), residual primary tumor with ypN0 (*n* = 119, 55.3%), and residual primary tumor with ypN+ (*n* = 49, 22.8%). Thus, among the 47 patients with complete regression of the primary tumor (ypT0), 13 (27.7%) had residual ypN-positive disease.

Among the 13 patients with ypT0N+ disease, 4 (30.8%) were classified as ypN1a, 5 (38.5%) as ypN1b, and 4 (30.8%) as ypN1c; none had ypN2 disease. Thus, 9 of 13 patients (69.2%) had metastatic regional lymph nodes, whereas 4 (30.8%) were classified as ypN1c on the basis of tumor deposits in the absence of positive lymph nodes. The median number of positive lymph nodes in the ypT0N+ group was 1 (IQR, 2.0; range, 0–3). Detailed nodal characteristics of both ypN-positive pathological response groups are provided in Supplementary Table S1 (Panel A).

### 3.2. Clinical, Treatment, and Pathological Characteristics

Baseline demographic and treatment-related characteristics according to pathological tumor–nodal response group are summarized in Table 1. Age, sex, American Society of Anesthesiologists (ASA) class, baseline serum CEA level, neoadjuvant treatment type, interval between neoadjuvant treatment and surgery, and operative approach did not differ significantly across the four groups. The median interval from completion of neoadjuvant treatment to surgery was 52 days (Q1–Q3, 45–58 days) in the overall cohort. The corresponding median intervals were 55 days (Q1–Q3, 51–61 days) after long-course chemoradiotherapy and 25 days (Q1–Q3, 20–31.5 days) after short-course radiotherapy. Pretreatment clinical T and N categories differed significantly among the pathological tumor–nodal response groups (both Fisher’s exact *p* < 0.001), with cT4 and cN2 disease concentrated predominantly in the residual primary tumor/ypN+ group. Completion of neoadjuvant treatment also differed across groups (Fisher’s exact *p* = 0.038), although treatment completion rates were high overall. The distribution of surgical procedures did not reach statistical significance (*p* = 0.053), and neither colorectal anastomosis nor stoma status differed significantly among groups. Adjuvant chemotherapy was administered substantially more frequently to patients with residual ypN-positive disease, particularly those in the ypT0N+ and residual primary tumor/ypN+ groups (*p* < 0.001). Among the 105 patients who received adjuvant chemotherapy, 39 (37.1%) received FOLFOX, 52 (49.5%) received CAPOX/XELOX, and 14 (13.3%) received capecitabine monotherapy. Regimen distributions according to pathological response group are provided in Supplementary Table S4.

Pathological characteristics are presented in Table 2. The median number of examined lymph nodes was comparable across groups (*p* = 0.518), as was the proportion of patients with a lymph-node yield of ≥12 (*p* = 0.315). In contrast, adverse pathological features were concentrated among patients with residual primary and nodal disease. Circumferential resection margin positivity was absent in both ypT0 groups but occurred in 18.5% of patients with residual primary tumor/ypN0 and 49.0% of those with residual primary tumor/ypN+ (*p* < 0.001). Histological subtype and tumor differentiation also differed significantly among the pathological response groups (both *p* < 0.001).

Among patients with residual primary tumors, lymphovascular invasion, perineural invasion, venous invasion, and tumor budding were all substantially more frequent in the ypN+ subgroup than in the ypN0 subgroup (all *p* < 0.001). These features were not assessed as comparative variables in patients with ypT0 because no residual primary tumor was available for their evaluation. Modified Ryan tumor regression grades were 0 in 47 patients (21.9%), 1 in 35 (16.3%), 2 in 88 (40.9%), and 3 in 45 (20.9%). Because the modified Ryan grading system reflects regression within the primary tumor bed and is structurally related to postoperative ypT status, no inferential comparison of Ryan grades across the four pathological tumor–nodal response groups was performed.

### 3.3. Recurrence Patterns and Oncological Outcomes

The median follow-up duration, estimated using the reverse Kaplan–Meier method, was 70.0 months (95% CI, 60.0–80.0 months). During follow-up, 43 patients (20.0%) developed local or distant recurrence. Distant metastases occurred in 38 patients (17.7%), whereas only seven patients (3.3%) experienced local recurrence. Eighty-two patients (38.1%) died during follow-up. Early postoperative mortality was 2.8% at 30 days (6/215) and 4.7% at 90 days (10/215). The distribution of oncological events according to pathological tumor–nodal response group is summarized in Table 3.

The ypT0N0 group had the most favorable recurrence profile, with recurrence occurring in 2 of 34 patients (5.9%) and distant metastasis in 1 patient (2.9%). In contrast, 6 of 13 patients with ypT0N+ disease (46.2%) developed recurrence, and all six experienced distant metastatic failure. Recurrence occurred in 17 of 119 patients (14.3%) with residual primary tumor/ypN0 and in 18 of 49 patients (36.7%) with residual primary tumor/ypN+, while distant metastasis occurred in 15 (12.6%) and 16 (32.7%) patients, respectively. Both recurrence and distant metastasis differed significantly across the four pathological response groups (both *p* < 0.001). Local recurrence remained uncommon, occurring in 1 of 34 (2.9%), 0 of 13 (0%), 4 of 119 (3.4%), and 2 of 49 (4.1%) patients, respectively, and did not differ significantly among groups (*p* = 1.000). When stratified by neoadjuvant treatment type, local recurrence occurred in 5 of 168 patients treated with long-course chemoradiotherapy (3.0%) and in 2 of 47 patients treated with short-course radiotherapy (4.3%), with no statistically significant difference between treatment groups (Fisher’s exact test, *p* = 0.649). Given the small total number of local recurrence events (*n* = 7), this comparison was considered descriptive.

Within the ypT0N+ subgroup, exploratory descriptive stratification showed recurrence and distant metastasis in 3 of 9 patients (33.3%) with metastatic regional lymph-node involvement (ypN1a/ypN1b) and in 3 of 4 patients (75.0%) with ypN1c disease; no inferential comparison was performed because of the very small subgroup sizes (Supplementary Table S1, Panel B).

### 3.4. Time to Recurrence and Time to Distant Metastasis

**Figure 2:**
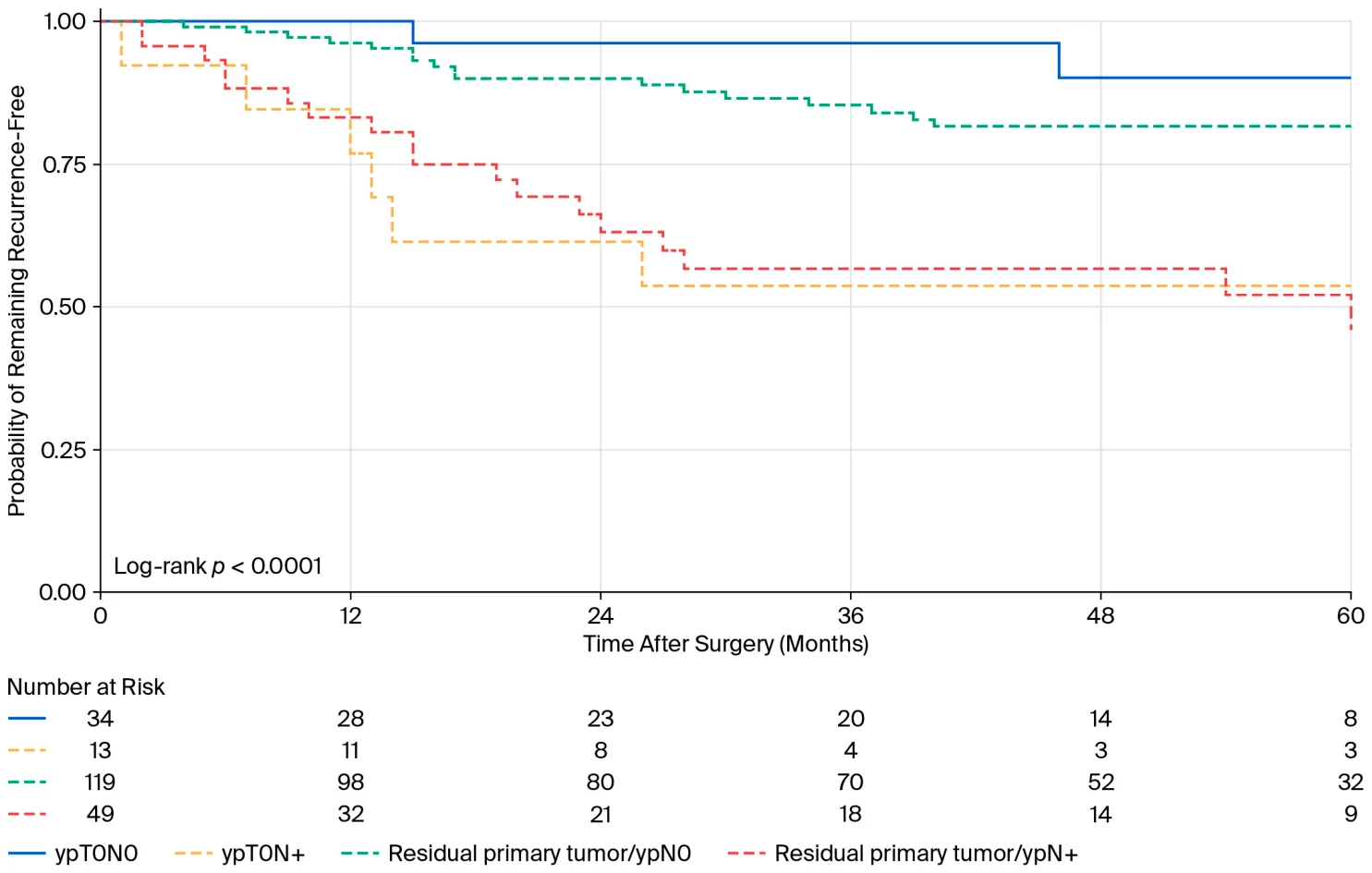
Kaplan–Meier curves for time to recurrence according to pathological tumor–nodal response group. Numbers at risk are shown below the plot. Differences among the four groups were assessed using the log-rank test (*p* < 0.0001).

Time to recurrence differed significantly among the four pathological tumor–nodal response groups (global log-rank *p* < 0.0001; Figure 2). The estimated probabilities of remaining recurrence-free at 5 years were 90.1% for ypT0N0, 53.8% for ypT0N+, 81.6% for residual primary tumor/ypN0, and 46.3% for residual primary tumor/ypN+. Pairwise comparisons showed significantly shorter TTR for ypT0N+ compared with ypT0N0 (Holm-adjusted *p* = 0.005) and residual primary tumor/ypN0 (Holm-adjusted *p* = 0.007). Residual primary tumor/ypN+ also had significantly shorter TTR than ypT0N0 (Holm-adjusted *p* = 0.003) and residual primary tumor/ypN0 (Holm-adjusted *p* < 0.001). In contrast, TTR did not differ significantly between ypT0N+ and residual primary tumor/ypN+ (Holm-adjusted *p* = 0.886).

**Figure 3:**
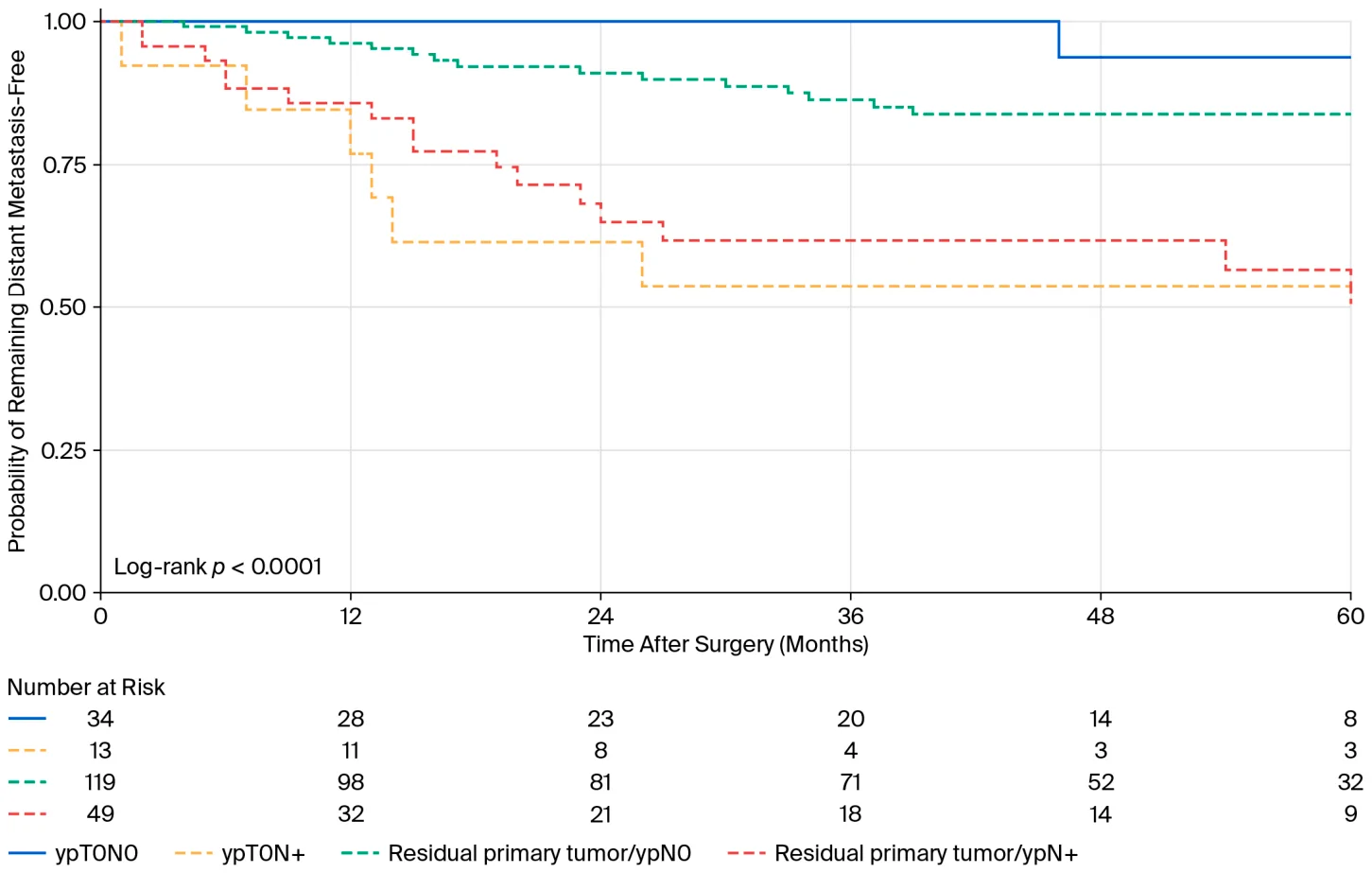
Kaplan–Meier curves for time to distant metastasis according to pathological tumor–nodal response group. Numbers at risk are shown below the plot. Differences among the four groups were assessed using the log-rank test (*p* < 0.0001).

Time to distant metastasis also differed significantly among the four groups (global log-rank *p* < 0.0001; Figure 3). The estimated probabilities of remaining free of distant metastasis at 5 years were 93.8% for ypT0N0, 53.8% for ypT0N+, 83.8% for residual primary tumor/ypN0, and 50.3% for residual primary tumor/ypN+. Pairwise comparisons demonstrated significantly shorter time to distant metastasis for ypT0N+ compared with ypT0N0 (Holm-adjusted *p* = 0.001) and residual primary tumor/ypN0 (Holm-adjusted *p* = 0.003). Residual primary tumor/ypN+ also had significantly shorter time to distant metastasis than ypT0N0 (Holm-adjusted *p* = 0.002) and residual primary tumor/ypN0 (Holm-adjusted *p* = 0.001). The difference between ypT0N+ and residual primary tumor/ypN+ was not statistically significant (Holm-adjusted *p* = 0.694).

Competing-risk analyses accounting for death without recurrence yielded concordant findings. The cumulative incidence of recurrence differed significantly across the four pathological tumor–nodal response groups (global Gray’s test *p* < 0.001). At 5 years, the cumulative incidence of recurrence was 7.9% for ypT0N0, 46.2% for ypT0N+, 15.3% for residual primary tumor/ypN0, and 40.5% for residual primary tumor/ypN+. Pairwise Gray tests showed significantly higher recurrence incidence for ypT0N+ compared with ypT0N0 and residual primary tumor/ypN0 (both Holm-adjusted *p* = 0.006), whereas ypT0N+ and residual primary tumor/ypN+ did not differ significantly (Holm-adjusted *p* = 0.466).

A similar pattern was observed for distant metastasis (global Gray’s test *p* < 0.001). The 5-year cumulative incidence of distant metastasis was 4.6% for ypT0N0, 46.2% for ypT0N+, 13.4% for residual primary tumor/ypN0, and 36.3% for residual primary tumor/ypN+. Pairwise comparisons demonstrated significantly higher cumulative incidence for ypT0N+ compared with ypT0N0 (Holm-adjusted *p* = 0.001) and residual primary tumor/ypN0 (Holm-adjusted *p* = 0.003), while the difference between ypT0N+ and residual primary tumor/ypN+ was not significant (Holm-adjusted *p* = 0.323). Detailed 3- and 5-year cumulative incidence estimates and pairwise Gray comparisons for recurrence and distant metastasis are provided in Supplementary Table S2 (Panels B and C).

### 3.5. Factors Associated with Time to Recurrence

Univariable Cox models identified baseline CEA, neoadjuvant treatment type, circumferential resection margin positivity, tumor differentiation, and adjuvant chemotherapy as variables associated with TTR; no such association was observed for age, lymph-node yield, the interval between neoadjuvant treatment and surgery, or surgical procedure (Table 4). The unfavorable univariable association observed for adjuvant chemotherapy should be viewed in light of treatment-by-indication imbalance, because postoperative therapy was used preferentially in patients with residual nodal disease.

In the primary adjusted Cox model, which included pathological tumor–nodal response group, neoadjuvant treatment type, age, and baseline CEA, recurrence risk differed according to pathological response pattern. Relative to ypT0N0, the recurrence hazard was higher in the ypT0N+ group (adjusted hazard ratio [aHR] 7.56, 95% CI 1.49–38.40; *p* = 0.015) and in patients with residual primary tumor/ypN+ (aHR 7.10, 95% CI 1.64–30.83; *p* = 0.009). No statistically significant difference was observed between residual primary tumor/ypN0 and ypT0N0 (aHR 2.15, 95% CI 0.49–9.40; *p* = 0.307). Higher baseline CEA was also associated with greater recurrence risk (aHR 1.07 per 1 ng/mL increase, 95% CI 1.03–1.11; *p* < 0.001), whereas neither age nor neoadjuvant treatment type reached statistical significance. The model was significant overall (likelihood-ratio χ^2^ = 33.77, df = 6; *p* < 0.001), and the global Schoenfeld test did not indicate departure from the proportional hazards assumption (*p* = 0.50).

Reparameterization of the model using residual primary tumor/ypN0 as the reference group showed higher recurrence hazards for both ypT0N+ (aHR 3.51, 95% CI 1.35–9.13; *p* = 0.010) and residual primary tumor/ypN+ (aHR 3.30, 95% CI 1.69–6.44; *p* < 0.001). Results from Firth penalized Cox regression were consistent with the primary analysis: compared with ypT0N0, the penalized HR was 6.62 for ypT0N+ (95% CI 1.64–36.72; *p* = 0.008) and 5.84 for residual primary tumor/ypN+ (95% CI 1.83–29.31; *p* = 0.002).

Further adjustment for pretreatment clinical T category yielded a similar estimate for ypT0N+ (aHR 7.72, 95% CI 1.52–39.12; *p* = 0.014), whereas the estimate for residual primary tumor/ypN+ was attenuated (aHR 3.79, 95% CI 0.81–17.71; *p* = 0.090). Pretreatment cT4 disease was associated with a higher recurrence hazard than cT3 disease in this model (aHR 3.45, 95% CI 1.63–7.30; *p* = 0.001). However, the proportional-hazards assumption was violated for pretreatment clinical T category; therefore, this model was considered a supportive sensitivity analysis rather than a primary basis for inference. The association for ypT0N+ was also retained when baseline CEA was entered after natural-log transformation (aHR 7.98, 95% CI 1.57–40.70; *p* = 0.012). Additional and sensitivity Cox model results are presented in Supplementary Table S2 (Panel A).

To further address potential confounding by pretreatment clinical stage, the primary model was repeated after restricting the analysis to patients with baseline cT3N1 disease (*n* = 161; 21 recurrence events). Within this baseline-stage–homogeneous subgroup, ypT0N+ remained associated with a higher recurrence hazard compared with ypT0N0 (aHR 6.34, 95% CI 1.19–33.79; *p* = 0.031). The corresponding estimates were 1.12 (95% CI 0.23–5.45; *p* = 0.884) for residual primary tumor/ypN0 and 4.27 (95% CI 0.83–21.87; *p* = 0.081) for residual primary tumor/ypN+. No meaningful violation of the proportional-hazards assumption was identified in this restricted model. Full results are presented in Supplementary Table S2 (Panel E).

Because CRM positivity was strongly associated with recurrence in univariable analysis, an additional sensitivity analysis was performed after excluding all CRM-positive patients. The CRM-negative cohort included 169 patients and 24 recurrence events. Compared with ypT0N0, ypT0N+ remained associated with a higher recurrence hazard (aHR 7.37, 95% CI 1.42–38.33; *p* = 0.018), as did residual primary tumor/ypN+ (aHR 5.56, 95% CI 1.15–26.81; *p* = 0.032). No meaningful violation of the proportional-hazards assumption was identified in this restricted analysis. Full results are presented in Supplementary Table S2 (Panel F).

Restricting the analysis to patients with a lymph-node yield of ≥12 left 157 patients and 32 recurrence events. Estimates generally remained directionally consistent with those from the primary model. For ypT0N+, the adjusted HR remained elevated but was estimated with substantial imprecision and did not reach statistical significance (aHR 6.16, 95% CI 0.66–57.13; *p* = 0.110). In contrast, residual primary tumor/ypN+ retained a statistically significant association with recurrence (aHR 8.00, 95% CI 1.03–61.97; *p* = 0.047). The global Schoenfeld test again supported the proportional hazards assumption (*p* = 0.55). Full results of this analysis are reported in Supplementary Table S2 (Panel D).

### 3.6. Overall Survival

**Figure 4:**
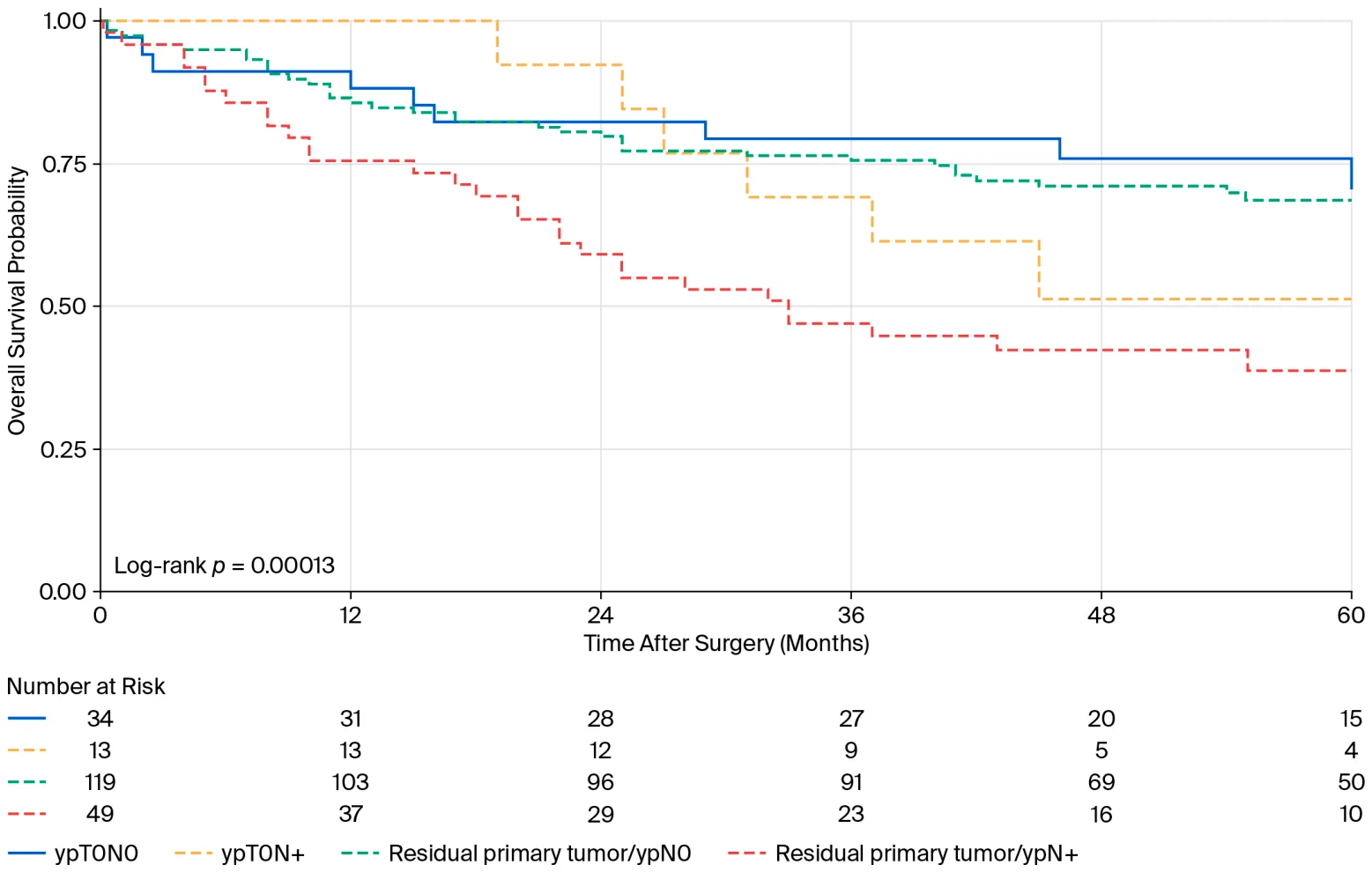
Kaplan–Meier curves for overall survival according to pathological tumor–nodal response group. Numbers at risk are shown below the plot. Differences among the four groups were assessed using the log-rank test (*p* = 0.00013).

Overall survival varied significantly across the four pathological tumor–nodal response groups (global log-rank *p* = 0.00013; Figure 4). The corresponding 5-year OS estimates were 70.9% for ypT0N0, 51.3% for ypT0N+, 68.7% for residual primary tumor/ypN0, and 38.8% for residual primary tumor/ypN+. In pairwise testing, patients with residual primary tumor/ypN+ had poorer OS than those with ypT0N0 (Holm-adjusted *p* = 0.008) and residual primary tumor/ypN0 (Holm-adjusted *p* < 0.001). No significant difference was observed between ypT0N0 and ypT0N+ (Holm-adjusted *p* = 0.757) or between ypT0N+ and residual primary tumor/ypN+ (Holm-adjusted *p* = 0.685).

In the adjusted Cox model containing pathological tumor–nodal response group, neoadjuvant treatment type, age, and baseline CEA, the residual primary tumor/ypN+ group had a higher mortality hazard than ypT0N0 (aHR 2.16, 95% CI 1.00–4.63; *p* = 0.049). By comparison, neither ypT0N+ (aHR 1.11, 95% CI 0.39–3.20; *p* = 0.845) nor residual primary tumor/ypN0 (aHR 0.93, 95% CI 0.44–1.94; *p* = 0.839) differed significantly from ypT0N0. Mortality hazard also increased with age (aHR 1.04 per year, 95% CI 1.01–1.07; *p* = 0.006) and baseline CEA concentration (aHR 1.06 per 1 ng/mL increase, 95% CI 1.03–1.09; *p* < 0.001), whereas neoadjuvant treatment type was not statistically significant. The global Schoenfeld test supported the proportional hazards assumption for this model (*p* = 0.149).

After exclusion of the 10 patients who died within 90 days of surgery, the sensitivity cohort comprised 205 patients with 72 deaths. In this restricted analysis, residual primary tumor/ypN+ again showed a higher mortality hazard relative to ypT0N0 (aHR 3.12, 95% CI 1.27–7.68; *p* = 0.013). The estimates for ypT0N+ (aHR 1.62, 95% CI 0.51–5.17; *p* = 0.411) and residual primary tumor/ypN0 (aHR 1.18, 95% CI 0.49–2.87; *p* = 0.711) did not reach statistical significance. The proportional hazards assumption remained supported in the restricted model (global Schoenfeld test *p* = 0.207). Full results of this sensitivity analysis are reported in Supplementary Table S3.

## 4. Discussion

The present study suggests that pathological response after neoadjuvant treatment for rectal cancer may be more appropriately interpreted as a combined tumor–nodal phenomenon rather than through primary-tumor regression alone. Although complete regression of the rectal primary was associated with excellent disease control in the absence of residual nodal disease, this favorable pattern was not preserved when residual ypN-positive disease persisted. Conversely, patients with residual primary tumor but no nodal involvement showed a substantially more favorable oncological course than those with persistent nodal disease. These findings indicate that nodal status modifies the prognostic meaning of primary-tumor response and may identify clinically relevant risk heterogeneity that is obscured when post-neoadjuvant response is summarized solely by the degree of regression within the primary tumor. The principal contribution of this response-based framework is therefore not limited to identifying the adverse prognosis of ypT0N+ disease, but extends to demonstrating how residual nodal disease alters the prognostic interpretation of primary-tumor response across distinct pathological response states. The particularly strong association of residual ypN-positive disease with recurrence and distant metastatic failure further suggests that discordant tumor–nodal response may serve as a pathological marker of differential treatment sensitivity and persistent metastatic potential, with implications for postoperative risk stratification.

The unfavorable course observed in patients with ypT0N+ disease is consistent with previous evidence indicating that complete primary-tumor regression does not neutralize the adverse prognostic effect of persistent nodal involvement. Multicenter and population-based studies have shown that ypT0N+ patients experience inferior disease control and survival compared with ypT0N0, while their outcomes may overlap with those of other node-positive post-neoadjuvant categories [7,8,9]. This pattern is also supported by earlier work demonstrating that residual nodal disease can outweigh an otherwise favorable primary-tumor response when tumor and nodal regression are discordant [12]. More recent series have reinforced this concept: ypN positivity remained associated with poorer disease-free survival among patients with ypT0 rectal cancer [10,11,21], and residual lymph-node metastasis continued to identify a higher-risk subgroup even among patients with marked primary-tumor downstaging to ypT0–2 [22]. Complementary analyses of lymph-node regression and combined primary-tumor regression grade and ypN status further suggest that nodal response provides prognostic information that is not fully captured by assessment of primary-tumor regression alone [16,18]. Taken together, these observations suggest that ypT0N+ should not be interpreted as an attenuated form of pathological complete response, but rather as a distinct post-neoadjuvant phenotype in which persistent nodal disease retains substantial prognostic relevance.

Although pairwise analyses did not demonstrate statistically significant differences between ypT0N+ and residual primary tumor/ypN+ for recurrence-related outcomes, this should not be interpreted as evidence of equivalence between these two groups. The small ypT0N+ subgroup and limited number of events substantially reduce the power to detect differences between two high-risk pathological phenotypes. Accordingly, the absence of statistical significance in these pairwise comparisons should be viewed as inconclusive rather than as evidence of comparable prognosis.

The proportion of residual nodal disease among patients with ypT0 in our cohort was 27.7%, underscoring that complete regression of the primary tumor does not invariably indicate eradication of regional disease. Reported frequencies of residual nodal involvement among patients with ypT0 vary across previous series and systematic reviews, potentially reflecting differences in baseline disease burden, case selection, neoadjuvant treatment protocols, pathological assessment, and the classification of residual nodal or deposit-related disease [7,8,21,23]. Given the relatively small number of patients with ypT0 in the present cohort, the observed proportion should be interpreted cautiously and warrants confirmation in larger contemporary datasets. The classification of tumor deposits is particularly relevant in this context because ypN1c disease contributed to the ypN-positive category in our cohort. Tumor deposits identified after neoadjuvant chemoradiotherapy have been associated with adverse oncological outcomes, while population-based data indicate that patients with N1c rectal cancer may have outcomes comparable to those with N1a–b disease [24,25]. In our exploratory subgroup analysis, recurrence occurred in 3 of 4 patients with ypN1c disease and in 3 of 9 patients with metastatic regional lymph-node involvement, with all recurrences in both subgroups involving distant metastasis. Although this descriptive pattern suggests that the unfavorable outcome of the ypT0N+ group was not confined to patients with metastatic regional lymph-node involvement, the very small number of ypN1c cases precludes reliable comparison or separate prognostic modeling according to the type of residual nodal disease.

Baseline CEA also remained independently associated with recurrence in the adjusted analysis. This finding is consistent with previous observations in patients with ypT0 rectal cancer, in whom elevated pretreatment CEA retained prognostic significance alongside residual nodal disease [21], and suggests that pathological response and pretreatment tumor biology may provide complementary prognostic information.

Pretreatment clinical T and N categories differed significantly across the pathological response groups, raising the possibility that baseline disease burden could confound the observed recurrence associations. To address this concern, we repeated the primary analysis after restricting the cohort to patients with pretreatment cT3N1 disease, the largest baseline-stage stratum represented across all four pathological response groups. Within this more homogeneous subgroup, ypT0N+ remained associated with a higher recurrence hazard than ypT0N0 (aHR 6.34, 95% CI 1.19–33.79; *p* = 0.031). An additional model adjusting for pretreatment clinical T category yielded a directionally similar estimate for ypT0N+; however, the proportional-hazards assumption was violated for clinical T category, and this model was therefore regarded as supportive rather than as a primary basis for inference. Taken together, these analyses reduce, but do not eliminate, the possibility that baseline clinical-stage imbalance accounts for the observed association, particularly given the limited number of recurrence events and the small ypT0N+ subgroup.

Neoadjuvant treatment type also warrants consideration when interpreting these findings. Short-course radiotherapy was numerically more frequent in the ypT0N+ (30.8%), residual primary tumor/ypN0 (21.8%), and residual primary tumor/ypN+ (26.5%) groups than in the ypT0N0 group (11.8%); however, the overall distribution of neoadjuvant treatment type did not differ significantly across the four pathological response groups (*p* = 0.353). Because treatment selection was non-randomized and the specific patient-level rationale for choosing long-course chemoradiotherapy or short-course radiotherapy could not be systematically reconstructed, these differences should not be interpreted as evidence of differential treatment efficacy. Local recurrence was uncommon after both approaches, occurring in 3.0% of patients treated with long-course chemoradiotherapy and 4.3% of those treated with short-course radiotherapy (*p* = 0.649). Given that only seven local recurrences occurred in the entire cohort, this comparison is descriptive and should not be interpreted as evidence of equivalent local control.

The recurrence pattern observed in the present cohort further supports the possibility that residual ypN-positive disease after neoadjuvant treatment reflects a broader propensity for systemic failure rather than merely persistent locoregional tumor burden. This distinction was particularly evident among patients with ypT0N+ disease, in whom all documented recurrences involved distant metastatic failure despite complete eradication of the primary tumor. Similar observations have been reported in contemporary rectal cancer series, in which post-neoadjuvant pathological stage and residual nodal involvement were closely linked to the risk and timing of distant recurrence, whereas local failure remained comparatively uncommon after adequate mesorectal surgery [14,26,27,28,29]. Importantly, the same prognostic hierarchy was preserved when death without recurrence was treated as a competing event, reducing the likelihood that differences in competing mortality alone accounted for the observed recurrence pattern. Such a pattern is biologically plausible, as viable residual tumor within the regional lymphatic compartment after an otherwise favorable primary-tumor response may represent a treatment-resistant subpopulation with continued metastatic potential. Importantly, however, nodal persistence should be regarded as a marker of adverse tumor biology rather than direct evidence of a causal pathway to systemic dissemination. Moreover, lymph-node yield was comparable across pathological response groups in our cohort, making differential nodal retrieval an unlikely explanation for the observed prognostic separation according to ypN status. A sensitivity analysis restricted to patients with a lymph-node yield of ≥12 yielded effect estimates in the same direction as the primary analysis; however, the ypT0N+ estimate was less precise and no longer statistically significant in the context of the reduced subgroup size and event count. From a clinical perspective, this distinction strengthens the rationale for incorporating nodal response into postoperative risk assessment, particularly when the primary tumor itself has completely regressed.

The pathological profile of patients with residual primary tumor and persistent nodal disease was also consistent with a more aggressive residual tumor phenotype. In our cohort, CRM positivity and several adverse microscopic features—including lymphovascular invasion, perineural invasion, venous invasion, and tumor budding—were substantially enriched in the residual primary tumor/ypN+ group. This clustering is clinically relevant because these features have each been associated with unfavorable oncological outcomes after neoadjuvant treatment and may reflect a common background of treatment-resistant residual disease rather than isolated pathological findings. In particular, tumor budding has emerged as a reproducible marker of adverse biology in neoadjuvantly treated rectal cancer, showing associations with nodal metastasis, lymphatic and venous invasion, perineural invasion, distant failure, and reduced survival [30,31]. Perineural invasion has likewise been linked to poorer prognosis in rectal cancer and appears to retain prognostic significance after preoperative treatment [32]. Taken together, the concordance between nodal persistence and these adverse pathological features in our series supports the view that postoperative ypN positivity is embedded within a broader high-risk biological phenotype. At the same time, the ypT0N+ subgroup remains particularly informative because its adverse outcome cannot be explained by residual primary-tumor morphology, underscoring the additional prognostic information carried by nodal status.

The relatively high CRM-positive rate in the present cohort warrants specific consideration. CRM positivity was observed in 46 of 215 patients (21.4%) overall and in 24 of 49 patients (49.0%) with residual primary tumor/ypN+ disease, and CRM positivity was strongly associated with recurrence in univariable analysis (HR 4.74, 95% CI 2.58–8.71; *p* < 0.001). Importantly, however, no CRM-positive cases were present in either ypT0 group. Furthermore, when all CRM-positive patients were excluded, the association between ypT0N+ disease and recurrence remained evident (aHR 7.37, 95% CI 1.42–38.33; *p* = 0.018). Thus, although CRM involvement represents an important adverse prognostic feature in this cohort, CRM positivity alone does not account for the adverse recurrence signal observed in the ypT0N+ subgroup.

Postoperative chemotherapy warrants cautious interpretation in this setting because treatment allocation is intrinsically linked to pathological risk. In our cohort, adjuvant chemotherapy was administered disproportionately to patients with residual ypN-positive disease, particularly those with ypT0N+ and residual primary tumor/ypN+ disease, yet these groups continued to experience less favorable recurrence-related outcomes. Among the 105 patients who received adjuvant chemotherapy, FOLFOX was used in 39 (37.1%), CAPOX/XELOX in 52 (49.5%), and capecitabine monotherapy in 14 (13.3%), with regimen distribution varying across the pathological response groups (Supplementary Table S4). Detailed information regarding completion of the planned postoperative chemotherapy course was not systematically available, precluding reliable evaluation of treatment completion as a modifier of oncological outcomes. This pattern should not be interpreted as evidence of limited treatment efficacy; rather, it illustrates the strong confounding by indication that accompanies retrospective comparisons of postoperative systemic therapy. Contemporary data suggest that adjuvant chemotherapy may improve disease control in ypN+ rectal cancer after neoadjuvant treatment, although the magnitude of benefit varies across pathological response strata and remains less certain in patients with favorable pathological downstaging [33,34,35]. In particular, a prospective multicenter cohort demonstrated lower systemic recurrence and improved disease-free survival among ypN+ patients receiving adjuvant chemotherapy, whereas population-based analyses have also reported survival advantages across both node-negative and node-positive post-neoadjuvant groups [33,34]. Conversely, evidence in patients achieving complete or near-complete pathological response remains heterogeneous, with meta-analyses showing discordant effects across overall survival, disease-free survival, and recurrence-related endpoints [35]. These uncertainties, together with the marked imbalance in treatment allocation in our series, support our decision not to include adjuvant chemotherapy in the primary multivariable model, where adjustment for a treatment determined after pathological assessment could have obscured rather than clarified the prognostic contribution of tumor–nodal response.

The overall survival findings were less pronounced than the recurrence-related endpoints and should therefore be interpreted with appropriate caution. Although OS differed significantly across the pathological tumor–nodal response groups, the separation between ypT0N0 and ypT0N+ was substantially less marked than that observed for TTR and time to distant metastasis. This is not unexpected, because overall survival integrates cancer-related mortality with postoperative deaths and competing non-cancer causes of death, thereby weakening the direct relationship between pathological response and subsequent tumor recurrence. Large population-based analyses have similarly shown that ypT0N+ disease may be associated with less favorable overall survival than ypT0N0, while its survival profile may overlap with other intermediate post-neoadjuvant pathological categories rather than following a simple stepwise gradient [8]. In the present cohort, ypT0N+ was not independently associated with OS after multivariable adjustment, whereas residual primary tumor/ypN+ remained associated with poorer survival. This association also persisted after exclusion of deaths occurring within 90 days after surgery. These findings further support the interpretation that recurrence-related endpoints may more directly reflect the prognostic implications of residual nodal disease and tumor biology, whereas OS represents a broader outcome influenced by both oncological and competing mortality processes [36].

From a clinical perspective, these findings argue against interpreting complete regression of the primary tumor as sufficient evidence of a uniformly favorable post-neoadjuvant prognosis. The distinction between ypT0N0 and ypT0N+ is particularly relevant because both groups share complete eradication of the rectal primary, yet their subsequent oncological trajectories diverge markedly once residual nodal disease is taken into account. Contemporary data similarly indicate that achievement of ypN0 is associated with substantially better long-term outcomes, whereas persistent nodal positivity identifies a group with sustained oncological risk despite otherwise favorable treatment response [10,13,14,37]. Accordingly, postoperative risk stratification should preserve nodal status as a distinct component of response assessment rather than subsuming ypT0N+ patients within a broader category of complete or near-complete responders. This distinction may be particularly relevant for informing risk-adapted surveillance discussions, postoperative multidisciplinary decision-making, and interpretation of the prognostic significance of an apparently complete primary-tumor response. Importantly, these findings should not be taken to imply that pathological response groups alone can determine postoperative management; rather, they support incorporating tumor–nodal response into a broader multidisciplinary assessment of residual risk.

Several limitations should be considered when interpreting these findings. First, the retrospective, single-center design introduces the possibility of selection bias, unmeasured confounding, and practice-specific effects that may limit external generalizability. In addition, the exclusion of patients for whom postoperative oncological outcomes could not be reliably ascertained may have introduced further selection bias. Second, the ypT0N+ subgroup was small (*n* = 13), and only 43 recurrence events occurred in the overall cohort, resulting in wide confidence intervals for several effect estimates and limiting the precision and complexity of multivariable modeling. Although sensitivity analyses restricted to patients with baseline cT3N1 disease and to CRM-negative patients supported the principal ypT0N+ association, these analyses reduce but cannot eliminate the possibility of residual confounding. The small number of ypN1c cases further precluded reliable separate prognostic modeling according to the form of residual ypN-positive disease.

Third, the study cohort largely reflects the period before the routine adoption of total neoadjuvant therapy at our institution. The upper limit of the inclusion period was intentionally selected to ensure at least 36 months of potential follow-up for recurrence-related outcomes in the most recently operated patients. Consequently, although this design provided mature oncological follow-up, the generalizability of our findings to contemporary patients treated with total neoadjuvant therapy remains to be established. Total neoadjuvant therapy has become an important component of contemporary rectal cancer management and has been associated with higher pathological complete response rates than conventional neoadjuvant chemoradiotherapy [1,38]. Analyses from the RAPIDO trial further indicate that patients achieving pathological complete response after total neoadjuvant therapy can experience favorable long-term oncological outcomes; however, these data do not establish whether discordant ypT0N+ disease carries a comparable prognostic significance in the total neoadjuvant therapy setting [39]. Whether the same degree of tumor–nodal discordance retains comparable prognostic significance after contemporary total neoadjuvant therapy therefore remains to be determined.

Additional limitations relate to pathological assessment and survival endpoints. Selected pathological variables, including lymphovascular invasion, perineural invasion, venous invasion, and tumor budding, could not be meaningfully assessed in ypT0 specimens because no residual primary tumor was present. Histological subtype and tumor differentiation in ypT0 cases were derived from pretreatment biopsy specimens, whereas these characteristics were assessed in resection specimens when residual primary tumor was present, which may have introduced some heterogeneity into pathological comparisons across response groups. Detailed information regarding completion of the planned postoperative chemotherapy course was not systematically available, precluding assessment of treatment completion as a potential modifier of oncological outcomes. Finally, overall survival was influenced by postoperative and non-cancer mortality, and cause-specific survival could not be evaluated consistently in all patients.

The adverse prognostic significance of residual nodal disease after neoadjuvant treatment has been demonstrated previously; therefore, the present study should be regarded as an incremental rather than a fundamentally novel contribution. Its principal contribution lies in the focused evaluation of the uncommon ypT0N+ phenotype within a four-group pathological response framework, together with mature follow-up and recurrence-focused sensitivity analyses.

These limitations are balanced by several strengths, including a well-defined consecutive cohort treated within a single multidisciplinary framework, detailed pathological characterization, explicit separation of primary-tumor and nodal response, and simultaneous evaluation of time to recurrence, time to distant metastasis, time to local recurrence, and overall survival. The four-group response framework also allowed the prognostic contribution of residual nodal disease to be examined both in the presence and in the complete absence of residual primary tumor, which represents a central methodological strength of the present study.

## 5. Conclusions

In this cohort, residual ypN-positive disease despite complete pathological regression of the primary rectal tumor was associated with an increased risk of recurrence, suggesting that primary-tumor response and nodal response provide complementary prognostic information after neoadjuvant treatment. These findings support considering residual nodal status separately when interpreting an apparently complete primary-tumor response. However, the small number of patients with ypT0N+ disease limits the precision of the effect estimates, and the present findings should be regarded as hypothesis-supporting until confirmed in larger independent cohorts.

## Figures and Tables

**Table 1:** Clinical, treatment, and surgical characteristics according to pathological tumor–nodal response groups.

Variable	Overall (*n* = 215)	Group 1 ypT0N0 (*n* = 34)	Group 2 ypT0N+ (*n* = 13)	Group 3 Residual Primary Tumor/ypN0 (*n* = 119)	Group 4 Residual Primary Tumor/ypN+ (*n* = 49)	*p* Value
Age, years, mean ± SD	65.5 ± 9.5	64.1 ± 7.2	68.8 ± 10.6	65.1 ± 9.7	66.9 ± 10.1	0.296
**Sex, *n* (%)**						**0.848**
Female	72 (33.5)	11 (32.4)	4 (30.8)	38 (31.9)	19 (38.8)	
Male	143 (66.5)	23 (67.6)	9 (69.2)	81 (68.1)	30 (61.2)	
**ASA score, *n* (%)**						**0.191 †**
ASA 2	90 (41.9)	19 (55.9)	5 (38.5)	52 (43.7)	14 (28.6)	
ASA 3	121 (56.3)	14 (41.2)	8 (61.5)	65 (54.6)	34 (69.4)	
ASA 4	4 (1.9)	1 (2.9)	0 (0.0)	2 (1.7)	1 (2.0)	
Baseline CEA, ng/mL, median (IQR)	4.25 (4.94)	4.18 (3.18)	3.11 (5.52)	4.14 (4.75)	5.62 (6.71)	0.158
**Pretreatment clinical T category, *n* (%)**						**<0.001 †**
cT3	169 (78.6)	34 (100.0)	13 (100.0)	97 (81.5)	25 (51.0)	
cT4	46 (21.4)	0 (0.0)	0 (0.0)	22 (18.5)	24 (49.0)	
**Pretreatment clinical N category, *n* (%)**						**<0.001 †**
cN0	18 (8.4)	0 (0.0)	0 (0.0)	18 (15.1)	0 (0.0)	
cN1	187 (87.0)	34 (100.0)	13 (100.0)	101 (84.9)	39 (79.6)	
cN2	10 (4.7)	0 (0.0)	0 (0.0)	0 (0.0)	10 (20.4)	
**Neoadjuvant treatment type, *n* (%)**						**0.353**
Long-course CRT	168 (78.1)	30 (88.2)	9 (69.2)	93 (78.2)	36 (73.5)	
Short-course RT	47 (21.9)	4 (11.8)	4 (30.8)	26 (21.8)	13 (26.5)	
**Neoadjuvant treatment completed, *n* (%)**						**0.038 †**
No	9 (4.2)	0 (0.0)	2 (15.4)	3 (2.5)	4 (8.2)	
Yes	206 (95.8)	34 (100.0)	11 (84.6)	116 (97.5)	45 (91.8)	
Interval from neoadjuvant treatment to surgery, days, median (IQR)	52 (13.0)	53.5 (10.8)	49 (8.0)	53 (11.5)	52 (22.0)	0.227
**Surgical approach, *n* (%)**						**0.278**
Open	180 (83.7)	26 (76.5)	11 (84.6)	98 (82.4)	45 (91.8)	
Laparoscopic	35 (16.3)	8 (23.5)	2 (15.4)	21 (17.6)	4 (8.2)	
**Surgical procedure, *n* (%)**						**0.053**
LAR	73 (34.0)	4 (11.8)	7 (53.8)	43 (36.1)	19 (38.8)	
uLAR	83 (38.6)	19 (55.9)	2 (15.4)	46 (38.7)	16 (32.7)	
APR	59 (27.4)	11 (32.4)	4 (30.8)	30 (25.2)	14 (28.6)	
**Colorectal anastomosis, *n* (%)**						**0.711**
No	66 (30.7)	11 (32.4)	4 (30.8)	33 (27.7)	18 (36.7)	
Yes	149 (69.3)	23 (67.6)	9 (69.2)	86 (72.3)	31 (63.3)	
**Stoma status, *n* (%)**						**0.529 †**
No stoma	69 (32.1)	8 (23.5)	6 (46.2)	38 (31.9)	17 (34.7)	
Loop ileostomy	80 (37.2)	15 (44.1)	3 (23.1)	48 (40.3)	14 (28.6)	
End colostomy	66 (30.7)	11 (32.4)	4 (30.8)	33 (27.7)	18 (36.7)	
**Adjuvant chemotherapy, *n* (%)**						**<0.001**
No	110 (51.2)	32 (94.1)	2 (15.4)	71 (59.7)	5 (10.2)	
Yes	105 (48.8)	2 (5.9)	11 (84.6)	48 (40.3)	44 (89.8)	

Data are presented as mean ± SD, median (IQR), or *n* (%), as appropriate. Residual primary tumor denotes ypTis–ypT4. The ypN+ category includes ypN1c cases classified on the basis of tumor deposits in the absence of metastatic regional lymph nodes. Continuous variables were compared using one-way ANOVA for normally distributed data and the Kruskal–Wallis test for non-normally distributed data. Categorical variables were compared using Pearson’s chi-square test or Fisher’s exact test, as appropriate. † Fisher’s exact test. SD, standard deviation; IQR, interquartile range; ASA, American Society of Anesthesiologists physical status classification; CEA, carcinoembryonic antigen; CRT, chemoradiotherapy; RT, radiotherapy; ANOVA, analysis of variance; LAR, low anterior resection; uLAR, ultra-low anterior resection; APR, abdominoperineal resection.

**Table 2:** Pathological characteristics according to pathological tumor–nodal response groups.

Variable	Overall (*n* = 215)	Group 1 ypT0N0 (*n* = 34)	Group 2 ypT0N+ (*n* = 13)	Group 3 Residual Primary Tumor/ypN0 (*n* = 119)	Group 4 Residual Primary Tumor/ypN+ (*n* = 49)	*p* Value
Nodes examined, median (IQR)	13 (6)	13.0 (6.75)	13.0 (3.00)	13.0 (3.00)	14.0 (7.00)	0.518
**LN yield, *n* (%)**						**0.315**
<12 nodes	58 (27.0)	13 (38.2)	2 (15.4)	29 (24.4)	14 (28.6)	
≥12 nodes	157 (73.0)	21 (61.8)	11 (84.6)	90 (75.6)	35 (71.4)	
**CRM status, *n* (%)**						**<0.001 †**
Negative	169 (78.6)	34 (100.0)	13 (100.0)	97 (81.5)	25 (51.0)	
Positive	46 (21.4)	0 (0.0)	0 (0.0)	22 (18.5)	24 (49.0)	
**Histologic type, *n* (%)**						**<0.001 †**
Conventional adenocarcinoma	184 (85.6)	34 (100.0)	13 (100.0)	103 (86.6)	34 (69.4)	
Mucinous adenocarcinoma	29 (13.5)	0 (0.0)	0 (0.0)	16 (13.4)	13 (26.5)	
Signet-ring cell carcinoma	2 (0.9)	0 (0.0)	0 (0.0)	0 (0.0)	2 (4.1)	
**Tumor differentiation, *n* (%)**						**<0.001 †**
Well differentiated	71 (33.0)	25 (73.5)	3 (23.1)	33 (27.7)	10 (20.4)	
Moderately differentiated	128 (59.5)	7 (20.6)	8 (61.5)	81 (68.1)	32 (65.3)	
Poorly differentiated	16 (7.4)	2 (5.9)	2 (15.4)	5 (4.2)	7 (14.3)	
**Modified Ryan tumor regression grade, *n* (%) §**						**—**
Grade 0	47 (21.9)	—	—	—	—	—
Grade 1	35 (16.3)	—	—	—	—	—
Grade 2	88 (40.9)	—	—	—	—	—
Grade 3	45 (20.9)	—	—	—	—	—
LVI positive, *n*/N (%) ‡	56/168 (33.3)	NA	NA	16/119 (13.4)	40/49 (81.6)	<0.001 †
PNI positive, *n*/N (%) ‡	34/168 (20.2)	NA	NA	11/119 (9.2)	23/49 (46.9)	<0.001 †
VI positive, *n*/N (%) ‡	20/168 (11.9)	NA	NA	5/119 (4.2)	15/49 (30.6)	<0.001 †
Tumor budding positive, *n*/N (%) ‡	58/168 (34.5)	NA	NA	31/119 (26.1)	27/49 (55.1)	<0.001 †

Data are presented as median (IQR) or *n* (%), as appropriate. Residual primary tumor denotes ypTis–ypT4. The ypN+ category includes ypN1c cases classified on the basis of tumor deposits in the absence of metastatic regional lymph nodes. Histologic type and tumor differentiation were obtained from the resection specimen when residual primary tumor was present; in ypT0 cases, these variables were derived from the pretreatment diagnostic biopsy. Continuous variables were compared using the Kruskal–Wallis test. Categorical variables were compared using Pearson’s chi-square test or Fisher’s exact test, as appropriate. † Fisher’s exact test. ‡ LVI, PNI, VI, and tumor budding were not assessable in ypT0 specimens; comparisons were therefore restricted to patients with residual primary tumor (Groups 3 and 4). § Modified Ryan tumor regression grade reflects treatment response within the primary tumor bed and is structurally related to postoperative ypT status; therefore, grades are presented descriptively for the overall cohort only and were not compared across the pathological tumor–nodal response groups. IQR, interquartile range; CRM, circumferential resection margin; LN, lymph node; LVI, lymphovascular invasion; PNI, perineural invasion; VI, venous invasion; NA, not assessable.

**Table 3:** Oncological events and Kaplan–Meier estimates according to pathological tumor–nodal response groups.

Outcome	Overall (*n* = 215)	Group 1 ypT0N0 (*n* = 34)	Group 2 ypT0N+ (*n* = 13)	Group 3 Residual Primary Tumor/ypN0 (*n* = 119)	Group 4 Residual Primary Tumor/ypN+ (*n* = 49)	*p* Value
**Recurrence, *n* (%)**	**43 (20.0)**	**2 (5.9)**	**6 (46.2)**	**17 (14.3)**	**18 (36.7)**	**<0.001**
3-year recurrence-free Kaplan–Meier estimate, % (95% CI)	—	96.2 (89.0–100.0)	53.8 (32.6–89.1)	85.4 (78.5–92.8)	56.8 (42.6–75.8)	<0.0001 ‡
5-year recurrence-free Kaplan–Meier estimate, % (95% CI)	—	90.1 (77.7–100.0)	53.8 (32.6–89.1)	81.6 (73.9–90.0)	46.3 (30.8–69.5)	
**Distant metastasis, *n* (%)**	**38 (17.7)**	**1 (2.9)**	**6 (46.2)**	**15 (12.6)**	**16 (32.7)**	**<0.001**
3-year distant-metastasis-free Kaplan–Meier estimate, % (95% CI)	—	100.0 (100.0–100.0)	53.8 (32.6–89.1)	86.4 (79.7–93.6)	61.8 (47.6–80.2)	<0.0001 ‡
5-year distant-metastasis-free Kaplan–Meier estimate, % (95% CI)	—	93.8 (82.6–100.0)	53.8 (32.6–89.1)	83.8 (76.6–91.8)	50.3 (34.1–74.2)	
**Local recurrence, *n* (%)**	**7 (3.3)**	**1 (2.9)**	**0 (0.0)**	**4 (3.4)**	**2 (4.1)**	**1.000 †**
**Death, *n* (%)**	**82 (38.1)**	**9 (26.5)**	**6 (46.2)**	**36 (30.3)**	**31 (63.3)**	**<0.001**
3-year OS, % (95% CI)	—	79.4 (66.9–94.2)	69.2 (48.2–99.5)	75.6 (68.3–83.8)	46.9 (34.9–63.2)	0.00013 ‡
5-year OS, % (95% CI)	—	70.9 (56.1–89.7)	51.3 (29.3–89.7)	68.7 (60.6–77.9)	38.8 (26.7–56.3)	

Data are presented as *n* (%) or Kaplan–Meier estimates with 95% confidence intervals, as appropriate. Group 1: ypT0N0; Group 2: ypT0N+; Group 3: residual primary tumor (ypTis–T4) with ypN0; Group 4: residual primary tumor (ypTis–T4) with ypN+. The ypN+ category includes ypN1c cases classified on the basis of tumor deposits in the absence of metastatic regional lymph nodes. TTR was defined as the interval from surgery to the first documented local or distant recurrence. Time to distant metastasis was defined as the interval from surgery to the first documented distant metastatic event. Time to local recurrence was defined as the interval from surgery to the first documented pelvic/local recurrence. OS was defined as the interval from surgery to death from any cause. Kaplan–Meier estimates for TTR and time to distant metastasis are expressed as the proportions remaining free of recurrence and distant metastasis, respectively. † Fisher’s exact test was used because of the low number of local recurrence events. ‡ Global comparison of Kaplan–Meier curves using the log-rank test. Pairwise log-rank comparisons were adjusted using the Holm method. CI, confidence interval; TTR, time to recurrence; OS, overall survival.

**Table 4:** Univariable and primary multivariable Cox proportional hazards regression analyses for time to recurrence.

Variable	Univariable HR (95% CI)	*p* Value	Primary Multivariable Model, Adjusted HR (95% CI)	*p* Value
**Pathological tumor–nodal response group**				
Group 1: ypT0N0	Reference	—	Reference	—
Group 2: ypT0N+	9.35 (1.88–46.37)	0.006	7.56 (1.49–38.40)	0.015
Group 3: residual primary tumor/ypN0	2.43 (0.56–10.53)	0.235	2.15 (0.49–9.40)	0.307
Group 4: residual primary tumor/ypN+	8.46 (1.96–36.48)	0.004	7.10 (1.64–30.83)	0.009
Age, per 1-year increase	1.02 (0.98–1.05)	0.322	1.01 (0.97–1.04)	0.772
Baseline CEA, per 1 ng/mL increase	1.06 (1.03–1.10)	<0.001	1.07 (1.03–1.11)	<0.001
**Neoadjuvant treatment type**				
Long-course CRT	Reference	—	Reference	—
Short-course RT	2.15 (1.10–4.20)	0.026	1.73 (0.82–3.65)	0.150
Interval from neoadjuvant treatment to surgery, per 1-day increase	0.983 (0.963–1.003)	0.093	—	—
**Surgical procedure**				
LAR	Reference	—	—	—
uLAR	0.56 (0.27–1.16)	0.121	—	—
APR	0.85 (0.41–1.76)	0.665	—	—
CRM status, positive vs. negative	4.74 (2.58–8.71)	<0.001	—	—
**Tumor differentiation**				
Well differentiated	Reference	—	—	—
Moderately differentiated	4.56 (1.79–11.65)	0.002	—	—
Poorly differentiated	3.84 (0.92–16.12)	0.066	—	—
Adjuvant chemotherapy, yes vs. no	4.74 (2.20–10.21)	<0.001	—	—
LN yield ≥ 12 vs. <12	1.10 (0.55–2.18)	0.787	—	—

Notes. HRs were estimated using Cox proportional hazards regression. The primary multivariable TTR model included pathological tumor–nodal response group, age, baseline carcinoembryonic antigen (CEA), and neoadjuvant treatment type. The model was specified to estimate the adjusted association between pathological tumor–nodal response and recurrence while limiting model instability and avoiding inappropriate adjustment for variables downstream of pathological response. CRM status and tumor differentiation were evaluated in univariable analyses but were not included in the primary adjusted model because of their close relationship with postoperative pathological response. Adjuvant chemotherapy was excluded from the primary model because treatment allocation occurred after pathological assessment and was strongly influenced by pathological risk, creating substantial potential for confounding by indication. No evidence of violation of the proportional hazards assumption was identified for the final TTR model based on Schoenfeld residuals (global *p* = 0.50). Group definitions: Group 1, ypT0N0; Group 2, ypT0N+; Group 3, residual primary tumor (ypTis–T4) with ypN0; Group 4, residual primary tumor (ypTis–T4) with ypN+. The ypN+ category includes ypN1c cases classified on the basis of tumor deposits in the absence of metastatic regional lymph nodes. CI, confidence interval; HR, hazard ratio; aHR, adjusted hazard ratio; CEA, carcinoembryonic antigen; CRT, chemoradiotherapy; RT, radiotherapy; CRM, circumferential resection margin; LN, lymph node; TTR, time to recurrence; LAR, low anterior resection; uLAR, ultra-low anterior resection; APR, abdominoperineal resection.

## Data Availability

The data presented in this study are available from the corresponding author upon reasonable request. The data are not publicly available due to patient privacy and ethical considerations.

## Supplementary Materials

The following supporting information can be downloaded at https://www.mdpi.com/article/10.3390/cancers18203352/s1, Table S1: Detailed nodal characteristics and exploratory outcomes in patients with residual ypN-positive disease; Table S2: Additional and sensitivity analyses for time to recurrence and competing-risk outcomes; Table S3: Sensitivity analysis of overall survival after exclusion of deaths within 90 days after surgery; Table S4: Distribution of adjuvant chemotherapy regimens according to pathological tumor–nodal response group.

## Abbreviations

The following abbreviations are used in this manuscript:

aHR	Adjusted hazard ratio
ANOVA	Analysis of variance
APR	Abdominoperineal resection
ASA	American Society of Anesthesiologists physical status classification
CAPOX/XELOX	Capecitabine plus oxaliplatin
CEA	Carcinoembryonic antigen
CI	Confidence interval
cM	Clinical metastasis category
cN	Clinical nodal category
CRM	Circumferential resection margin
CRT	Chemoradiotherapy
cT	Clinical tumor category
FOLFOX	Folinic acid, fluorouracil, and oxaliplatin
HR	Hazard ratio
IQR	Interquartile range
LAR	Low anterior resection
LN	Lymph node
LVI	Lymphovascular invasion
MRI	Magnetic resonance imaging
NA	Not assessable
OS	Overall survival
PNI	Perineural invasion
RT	Radiotherapy
SD	Standard deviation
TTR	Time to recurrence
uLAR	Ultra-low anterior resection
VI	Venous invasion
ypN	Post-neoadjuvant pathological nodal category
ypT	Post-neoadjuvant pathological tumor category
ypTNM	Post-neoadjuvant pathological tumor–node–metastasis classification
