# Supplementary material for: Pathological Tumor–Nodal Response Patterns After Neoadjuvant Treatment for Rectal Cancer: Prognostic Impact of Residual ypN-Positive Disease
Source: Cancers (Basel). 2026 Oct 16;18(20):3352. doi: 10.3390/cancers18203352 (PMC13702116; doi:10.3390/cancers18203352)
Supplement: Supplementary file 1 [file cancers-18-03352-s001.zip › cancers-4614055-supplementary.pdf]

Supplementary Table S1. Detailed nodal characteristics and exploratory outcomes in patients with residual ypN-positive disease

Panel A. Detailed nodal characteristics among patients with residual ypN-positive disease

Variable	ypT0N+ (n = 13)	Residual primary tumor/ypN+ (n = 49)
**ypN category, n (%)**		
ypN1a	4 (30.8)	10 (20.4)
ypN1b	5 (38.5)	9 (18.4)
ypN1c	4 (30.8)	20 (40.8)
ypN2a	0 (0.0)	5 (10.2)
ypN2b	0 (0.0)	5 (10.2)
Metastatic regional lymph nodes present, n (%)	9 (69.2)	29 (59.2)
Positive lymph nodes, median (IQR)	1 (2.0)	1 (2.0)
Positive lymph nodes, range	0–3	0–15

Note. Data are presented as n (%) or median (interquartile range), as appropriate. ypN1c denotes tumor deposits in the absence of metastatic regional lymph nodes. The number of positive lymph nodes includes zero values for ypN1c cases. No inferential comparisons were performed because these variables describe the nodal composition of the ypN-positive pathological response groups. IQR, interquartile range.

Panel B. Exploratory oncological outcomes within the ypT0N+ subgroup according to type of residual nodal disease

Residual nodal disease type	n	Recurrence, n (%)	Distant metastasis, n (%)
Metastatic regional lymph-node involvement (ypN1a/ypN1b)	9	3 (33.3)	3 (33.3)
ypN1c (tumor deposits without metastatic regional lymph nodes)	4	3 (75.0)	3 (75.0)

Note. Data are presented descriptively. Because of the very small number of ypN1c cases, no separate prognostic modeling according to the type of residual nodal disease was performed. All recurrences observed within the ypT0N+ subgroup involved distant metastasis.

Supplementary Table S2. Additional and sensitivity analyses for time to recurrence and competing-risk outcomes

Panel A. Additional and sensitivity Cox analyses for time to recurrence

A1. Reparameterized primary Cox model

Pathological tumor–nodal response group	Adjusted HR (95% CI)	p value
Residual primary tumor/ypN0	Reference	—
ypT0N0	0.46 (0.11–2.02)	0.307
ypT0N+	3.51 (1.35–9.13)	0.010
Residual primary tumor/ypN+	3.30 (1.69–6.44)	<0.001

The reference category was changed to residual primary tumor/ypN0; covariates were otherwise identical to the primary model.

A2. Firth penalized Cox regression

Pathological tumor–nodal response group	Penalized HR (95% CI)	p value
ypT0N0	Reference	—
ypT0N+	6.62 (1.64–36.72)	0.008
Residual primary tumor/ypN0	1.80 (0.56–9.06)	0.354
Residual primary tumor/ypN+	5.84 (1.83–29.31)	0.002

The penalized model included pathological tumor–nodal response group, neoadjuvant treatment type, age, and baseline CEA.

A3. Sensitivity model additionally adjusted for pretreatment clinical T category

Variable	Adjusted HR (95% CI)	p value
ypT0N0	Reference	—
ypT0N+	7.72 (1.52–39.12)	0.014
Residual primary tumor/ypN0	1.62 (0.36–7.23)	0.528
Residual primary tumor/ypN+	3.79 (0.81–17.71)	0.090
cT4 vs cT3	3.45 (1.63–7.30)	0.001

The model additionally included pretreatment clinical T category and otherwise retained neoadjuvant treatment type, age, and baseline CEA. The proportional-hazards assumption was violated for pretreatment clinical T category (Schoenfeld $p < 0.001$; global test $p = 0.002$); therefore, this model was interpreted as a supportive sensitivity analysis rather than as a primary basis for inference.

A4. Sensitivity model using natural-log-transformed baseline CEA

Variable	Adjusted HR (95% CI)	p value
ypT0N0	Reference	—
ypT0N+	7.98 (1.57–40.70)	0.012
Residual primary tumor/ypN0	2.43 (0.56–10.53)	0.236
Residual primary tumor/ypN+	6.81 (1.57–29.62)	0.011
ln(CEA), per 1-unit increase	1.82 (1.23–2.70)	0.003

The model replaced baseline CEA with its natural logarithm and otherwise retained pathological tumor–nodal response group, neoadjuvant treatment type, and age.

Panel B. Competing-risk analysis for recurrence

Pathological tumor–nodal response group	3-year cumulative incidence, %	5-year cumulative incidence, %
ypT0N0	3.3	7.9
ypT0N+	46.2	46.2
Residual primary tumor/ypN0	12.3	15.3
Residual primary tumor/ypN+	33.6	40.5

Global Gray's test for recurrence: $p < 0.001$.

Pairwise comparison	Holm-adjusted Gray p value
ypT0N0 vs ypT0N+	0.006
ypT0N0 vs residual primary tumor/ypN0	0.450
ypT0N0 vs residual primary tumor/ypN+	0.006
ypT0N+ vs residual primary tumor/ypN0	0.006
ypT0N+ vs residual primary tumor/ypN+	0.466
Residual primary tumor/ypN0 vs residual primary tumor/ypN+	0.006

Panel C. Competing-risk analysis for distant metastasis

Pathological tumor–nodal response group	3-year cumulative incidence, %	5-year cumulative incidence, %
ypT0N0	0.0	4.6
ypT0N+	46.2	46.2
Residual primary tumor/ypN0	11.4	13.4
Residual primary tumor/ypN+	29.3	36.3

Global Gray's test for distant metastasis: $p < 0.001$.

Pairwise comparison	Holm-adjusted Gray p value
ypT0N0 vs ypT0N+	0.001
ypT0N0 vs residual primary tumor/ypN0	0.247
ypT0N0 vs residual primary tumor/ypN+	0.006
ypT0N+ vs residual primary tumor/ypN0	0.003
ypT0N+ vs residual primary tumor/ypN+	0.323
Residual primary tumor/ypN0 vs residual primary tumor/ypN+	0.007

Panel D. Sensitivity analysis restricted to patients with a lymph-node yield of ≥12

Variable	Adjusted HR (95% CI)	p value
ypT0N0	Reference	—
ypT0N+	6.16 (0.66–57.13)	0.110
Residual primary tumor/ypN0	3.02 (0.39–23.15)	0.288
Residual primary tumor/ypN+	8.00 (1.03–61.97)	0.047
Short-course RT vs long-course CRT	1.73 (0.74–4.05)	0.204
Age, per 1-year increase	1.01 (0.97–1.05)	0.690
Baseline CEA, per 1 ng/mL increase	1.07 (1.03–1.11)	0.001

The analysis was restricted to patients with a lymph-node yield of ≥12 (n = 157; 32 recurrence events). The multivariable model included pathological tumor–nodal response group, neoadjuvant treatment type, age, and baseline CEA, identical to the primary TTR model. No evidence of violation of the proportional hazards assumption was identified based on Schoenfeld residuals (global p = 0.55).

Panel E. Sensitivity analysis restricted to patients with pretreatment cT3N1 disease

Variable	Adjusted HR (95% CI)	p value
ypT0N0	Reference	—
ypT0N+	6.34 (1.19–33.79)	0.031
Residual primary tumor/ypN0	1.12 (0.23–5.45)	0.884
Residual primary tumor/ypN+	4.27 (0.83–21.87)	0.081
Short-course RT vs long-course CRT	1.58 (0.59–4.23)	0.365
Age, per 1-year increase	1.02 (0.96–1.07)	0.595
Baseline CEA, per 1 ng/mL increase	1.11 (1.01–1.22)	0.033

The analysis was restricted to patients with pretreatment cT3N1 disease (n = 161; 21 recurrence events). The multivariable model included pathological tumor–nodal response group, neoadjuvant treatment type, age, and baseline CEA, identical to the primary TTR model. No evidence of meaningful violation of the proportional hazards assumption was identified based on Schoenfeld residuals.

Panel F. Sensitivity analysis restricted to CRM-negative patients

Variable	Adjusted HR (95% CI)	p value
ypT0N0	Reference	—
ypT0N+	7.37 (1.42–38.33)	0.018
Residual primary tumor/ypN0	1.31 (0.28–6.20)	0.730
Residual primary tumor/ypN+	5.56 (1.15–26.81)	0.032
Short-course RT vs long-course CRT	1.64 (0.62–4.34)	0.323
Age, per 1-year increase	0.99 (0.94–1.04)	0.697
Baseline CEA, per 1 ng/mL increase	1.09 (0.99–1.20)	0.082

The analysis was restricted to CRM-negative patients (n = 169; 24 recurrence events). The multivariable model included pathological tumor–nodal response group, neoadjuvant treatment type, age, and baseline CEA, identical to the primary TTR model. No meaningful violation of the proportional hazards assumption was identified based on Schoenfeld residuals.

Note. For competing-risk analyses, death without the respective oncological event was treated as a competing event. Cumulative incidence functions were compared using Gray's test, and pairwise comparisons were adjusted using the Holm procedure. The global Gray p values for the competing event of death without recurrence and death without distant metastasis were 0.400 and 0.226, respectively. The ypN+ category includes ypN1c cases classified on the basis of tumor deposits in the absence of metastatic regional lymph nodes. CEA, carcinoembryonic antigen; CI, confidence interval; HR, hazard ratio; TTR, time to recurrence.

Supplementary Table S3. Sensitivity analysis of overall survival after exclusion of deaths within 90 days after surgery

Sensitivity cohort: n = 205; deaths = 72. Patients who died within 90 postoperative days (n = 10) were excluded.

Variable	Univariable HR (95% CI)	p value	Adjusted HR (95% CI)	p value
**Pathological tumor–nodal response group**				
Group 1: ypT0N0	Reference	—	Reference	—
Group 2: ypT0N+	2.65 (0.85–8.22)	0.092	1.62 (0.51–5.17)	0.411
Group 3: residual primary tumor/ypN0	1.50 (0.62–3.59)	0.366	1.18 (0.49–2.87)	0.711
Group 4: residual primary tumor/ypN+	4.54 (1.88–10.96)	0.001	3.12 (1.27–7.68)	0.013
**Neoadjuvant treatment type**				
Long-course CRT	Reference	—	Reference	—
Short-course RT	2.72 (1.68–4.41)	<0.001	1.74 (1.00–3.03)	0.050
Age, per 1-year increase	1.06 (1.03–1.09)	<0.001	1.04 (1.01–1.08)	0.008
Baseline CEA, per 1 ng/mL increase	1.05 (1.03–1.08)	<0.001	1.07 (1.03–1.10)	<0.001

Note. HRs were estimated using Cox proportional hazards regression. The sensitivity analysis excluded all patients who died within 90 days after surgery. The adjusted model included pathological tumor–nodal response group, neoadjuvant treatment type, age, and baseline CEA. No evidence of violation of the proportional hazards assumption was identified in the 90-day mortality-excluded OS sensitivity model based on Schoenfeld residuals (global p = 0.207). Group definitions: Group 1, ypT0N0; Group 2, ypT0N+; Group 3, residual primary tumor (ypTis–T4) with ypN0; Group 4, residual primary tumor (ypTis–T4) with ypN+. The ypN+ category includes ypN1c cases classified on the basis of tumor deposits in the absence of metastatic regional lymph nodes. CI, confidence interval; HR, hazard ratio; CEA, carcinoembryonic antigen; CRT, chemoradiotherapy; RT, radiotherapy; OS, overall survival.

Supplementary Table S4. Distribution of adjuvant chemotherapy regimens according to pathological tumor–nodal response group

Pathological tumor–nodal response group	Total n	Adjuvant chemotherapy, n (%)	FOLFOX, n (% of treated)	CAPOX/XELOX, n (% of treated)	Capecitabine, n (% of treated)
Group 1: ypT0N0	34	2 (5.9%)	0 (0.0%)	1 (50.0%)	1 (50.0%)
Group 2: ypT0N+	13	11 (84.6%)	5 (45.5%)	5 (45.5%)	1 (9.1%)
Group 3: residual primary tumor/ypN0	119	48 (40.3%)	14 (29.2%)	25 (52.1%)	9 (18.8%)
Group 4: residual primary tumor/ypN+	49	44 (89.8%)	20 (45.5%)	21 (47.7%)	3 (6.8%)
**Overall**	**215**	**105 (48.8%)**	**39 (37.1%)**	**52 (49.5%)**	**14 (13.3%)**

Note. Percentages for FOLFOX, CAPOX/XELOX, and capecitabine were calculated among patients who received adjuvant chemotherapy within each pathological response group. Detailed information regarding completion of the planned postoperative chemotherapy course was not systematically available in the retrospective records. FOLFOX, folinic acid, fluorouracil, and oxaliplatin; CAPOX/XELOX, capecitabine and oxaliplatin.
